# Injectable non-leaching tissue-mimetic bottlebrush elastomers as an advanced platform for reconstructive surgery

**DOI:** 10.1038/s41467-021-23962-8

**Published:** 2021-06-25

**Authors:** Erfan Dashtimoghadam, Farahnaz Fahimipour, Andrew N. Keith, Foad Vashahi, Pavel Popryadukhin, Mohammad Vatankhah-Varnosfaderani, Sergei S. Sheiko

**Affiliations:** 1 Department of Chemistry, University of North Carolina at Chapel Hill, Chapel Hill, USA; 2 Division of Comprehensive Oral Health, Periodontology, Adams School of Dentistry, University of North Carolina at Chapel Hill, Chapel Hill, NC USA; 3 Institute of Macromolecular Compounds of the Russian Academy of Sciences, St. Petersburg, Russia

**Keywords:** Mechanical properties, Biomaterials, Bioinspired materials

## Abstract

Current materials used in biomedical devices do not match tissue’s mechanical properties and leach various chemicals into the body. These deficiencies pose significant health risks that are further exacerbated by invasive implantation procedures. Herein, we leverage the brush-like polymer architecture to design and administer minimally invasive injectable elastomers that cure in vivo into leachable-free implants with mechanical properties matching the surrounding tissue. This strategy allows tuning curing time from minutes to hours, which empowers a broad range of biomedical applications from rapid wound sealing to time-intensive reconstructive surgery. These injectable elastomers support in vitro cell proliferation, while also demonstrating in vivo implant integrity with a mild inflammatory response and minimal fibrotic encapsulation.

## Abstract (web summary)

Minimally invasive materials that match tissues mechanical properties are needed for biomedical applications. Here, the authors report on brush-like polymers which can be injected and cured in situ, resulting in elastomeric implants which mechanically blend with surrounding tissue and show minimal foreign body response.

## Introduction

Prevailing technologies for biomedical devices continually attempt to iterate upon polymer gels to improve their tissue-mimetic properties^1–5^. However, as bioengineering materials, gels inherently suffer from three critical limitations. First, significant mechanical mismatch between gels and surrounding tissue results in body disfigurement from capsular contracture and eventual gel fracture^6–9^. Second, chemical leaching from gels pose long-term health risks from autoimmune disorders to cancer, particularly from the organogels commonly used in plastic surgery^10–17^. Lastly, invasive implantation procedures entail post-operative scarring and inflammation^18,19^. To date, the development of minimally invasive solvent-free injectable implants that do not leach into the body and precisely replicate the deformation response of soft biological tissues remains elusive.

Biological tissue’s response to deformation is explicitly characterized by a unique combination of softness (initially low modulus of ~1 kPa) and firmness (~100-fold stiffening with deformation)^20–23^. Gel’s inability to replicate this mechanical combination is due to the inherent flexibility of their linear network strands. Although swelling with solvent reduces crosslink density to access tissue-soft materials, the relatively high strand flexibility precludes mimicking tissues strong strain-stiffening response (aka firmness) as network strands remain outside of their finite extensibility range^23^. As such, state-of-the-art gel-based implants such as for body reconstruction are soft, but not firm. This mechanical limitation was resolved by utilizing the brush-like polymer architecture where densely grafted side-chains concurrently dilute and stiffen network strands to enable elastomers with concurrently enhanced softness and firmness^21–25^. Independently controlling these mechanical characteristics without adding solvent as a mechanical regulator^22^ allows mimicking the stress-strain response of various tissues ranging from supersoft brain tissue to tough skin^26,27^. However, in vivo implementation of this technology is severely limited as such elastomer synthesis typically involves solvent and crosslinking schemes that requires hazardous stimuli such as temperature and UV light^22,26,28,29^. To mitigate these issues, we exploit two vital traits of the brush architecture: (i) compact molecular conformation providing low melt viscosity and (ii) a myriad of chain ends apt for functionalization. This enables solvent-free in vivo injection of reactive bottlebrush melts to yield non-leachable elastomers that match the mechanics of surrounding tissue. Depending on the targeted application, the developed methodology allows fine-tuning both the Young’s modulus from 10^2^ of adipose tissue to 10^5^ Pa of skin and the gelation time from hours to minutes to match the duration of various surgical procedures. Given the design-by-architecture approach, this technology can be translated into a broad range of chemical compositions to satisfy specific functionality and biocompatibility requirements of various applications including reconstructive surgery, regenerative medicine, drug-delivery, soft robotics, and wearable diagnostics. Specifically, injectable elastomers are an attractive alternative to invasive deployment of reconstructive implants as they offer improved patient comfort, reduced costs, faster recovery, and minimal surgical and post-surgical complications.

## Results

### Concept and synthesis of injectable solvent-free elastomers

**Fig. 1:**
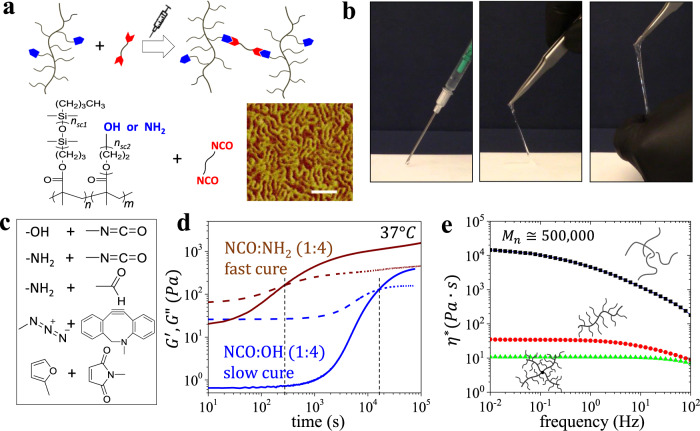
Synthesis of injectable non-leachable elastomers with tunable gelation time for biomedical applications. **a** Injectable tissue-mimetic elastomers composed of random polydimethylsiloxane-poly(ethylene glycol) (PDMS-*r*-PEG) brush copolymers with a controlled fraction of end-functionalized side-chains and a linear difunctional crosslinker. Inset: AFM micrograph of bottlebrush melt (${n}_{{sc}}$14, ${n}_{{bb}}$889) (sale bar 50 nm) shows densely packed worm-like macromolecules (Supplementary Fig. 12, Supplementary Table 3). **b** Demonstrating solvent-free injection and curing of a premixed dual component injectable formulation into an elastomer with a tissue-like deformation response (Supplementary Video 1). **c** Examples of coupling chemistries to crosslink functionalized bottlebrushes. **d** Evolution of the storage (G′) and loss (G″) moduli as a function of time for injectable elastomers with either OH-functionalized (slow-cure gelation, ${t}_{{gel}}$ ~ hours) or NH_2_-functionalized (fast-cure gelation, ${t}_{{gel}}$ ~ minutes) brush chain ends cured with a macromolecular diisocyanate crosslinker. **e** Polydimethylsiloxane (PDMS) melts with varying architecture (linear, bottlebrush, and star-like bottlebrush) and a similar molecular weight M_*n*_ ≅ 500,000 demonstrate significant decrease of zero-shear complex viscosity ($\eta \ast$) with branching. Further, bottlebrushes with longer side chains, yet similar molecular weights (${n}_{{sc}}$14, ${n}_{{bb}}$1540 *vs*. ${n}_{{sc}}$70, ${n}_{{bb}}$304) possess lower melt viscosity (Supplementary Fig. 13).

A dual syringe formulation consists of two reactive components: (i) a melt of bottlebrushes with functionalized side chains, and (ii) a difunctional crosslinker (Fig. 1a). Upon co-injecting, the functionalized bottlebrush and crosslinker mixture spontaneously reacts to yield a super-soft elastomer (Fig. 1b) with tunable mechanical properties by augmenting their stoichiometry as discussed below. Given the large size of bottlebrush macromolecules, a minuscule fraction of crosslinking moieties (0.02 mol%) is required to achieve a fully conjugated network, which minimizes uncontrolled reaction with surrounding tissue and precludes polymerization-induced shrinkage. To adjust the gelation time (${t}_{{gel}}$), a broad range of crosslinking chemistries has been considered including isocyanate/hydroxyl, isocyanate/amine, aldehyde/amine, alkyne/azide, and diene/dienophile (Fig. 1c)^30–34^. In this study, we primarily explore two systems for fast (${t}_{{gel}}$ ~ minutes) and slow (${t}_{{gel}}$ ~ hours) gelation rates respectively using isocyanate:hydroxyl (NCO:OH) and isocyanate:amine (NCO:NH_2_) coupling (Fig. 1d), yet other crosslinking schemes such as Diels-Alder chemistry can be considered if longer curing times are desired^35^. Fine-tuning ${t}_{{gel}}$ is also achieved by manipulating the fraction of functionalized chain ends, temperature, and catalyst concentration as discussed below. In all cases, solvent-free injection is empowered by a significant reduction of brush melt viscosity relative to linear polymers with identical molecular weight due to limited overlap and entanglement of bottlebrush macromolecules (Fig. 1e)^36^. Additional decreases in viscosity can be achieved by using more complex architectures such as star-like bottlebrush melts (Fig. 1e).

To enable injectable materials with tunable mechanics, adjustable curing rate, and an enhanced biocompatibility profile, it is imperative to design brushes with reactive moieties that meet critical criteria including: (i) targeted yet small fractions of functionalized reactive side chain ends with (ii) broad post-functionalization potential that are (iii) randomly dispersed throughout the brush, and which (iv) do not interact until curing. Although these features are readily programmed individually, taken together, they represent a significant synthetic challenge in respect to affording the desired mechanical properties, curing time, and biocompatibility profiles. To this end, bottlebrushes were synthesized by atom transfer radical polymerization (ATRP) of polydimethylsiloxane-methacrylate (PDMSMA) macromonomers with controlled fractions of polyethyleneglycol-methacrylate (PEGMA) macromonomers capped by either hydroxyl (OH-) or azide (N_3_-) ends (see “Methods” Section and Supplementary Figs. 1–8). Through ATRP, a random distribution of macromonomers was achieved as monitored and verified by time-resolved ^1^H-NMR (Supplementary Figs. 2 and 3). Atomic Force Microscopy (Fig. 1a) corroborates successful brush synthesis as depicted by imaging of worm-like macromolecules. In accordance with the time-resolved ^1^H-NMR of the macromonomer copolymerization, the resulting functional moieties are minuscule (~0.02 mol%), which hinders intermolecular interaction and enables adequate melt viscosity for injection as demonstrated by rheology (Fig. 1e). Finally, OH-functionalized brushes can be used as prepared or as precursors for further moieties (i.e., furan and methacrylate Supplementary Figs. 9–11, Supplementary Tables 1, 2), while NH_2_-functionalization was achieved through reduction of N_3_- chain ends (see “Methods” section and Supplementary Fig. 1), which highlights the potential for future tailored functional brushes for honing curing times and biocompatibility.

### Controlling gelation time of injectable elastomers

**Fig. 2:**
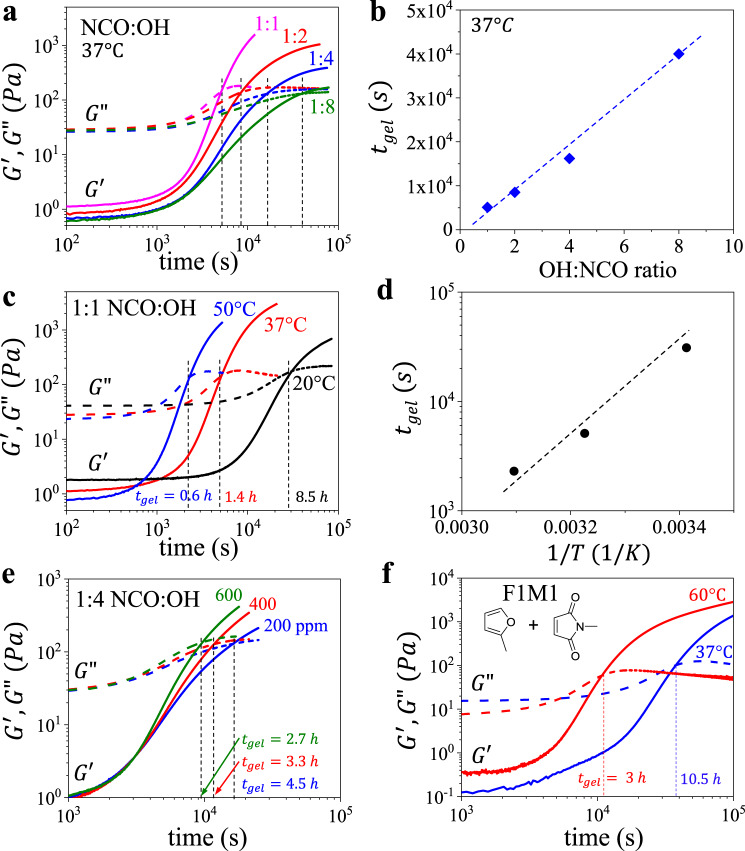
Gelation time of injectable elastomers. **a** Evolution of storage (G′) and loss (G″) moduli as a function of time for injectable elastomers comprising decreasing NCO:OH ratios (1:1, 2, 4, or 8). **b** Correlation of gelation time (${t}_{{gel}}$) and NCO:OH ratio. **c** Evolution of G′ and G″ as a function of time for injectable elastomer NCO:OH 1:1 at increasing temperatures of 20, 37, and 50 °C. **d** Correlation of gelation time (${t}_{{gel}}$) and temperature for injectable elastomer NCO:OH 1:1. **e** Evolution of G′ and G″ as a function of time for injectable elastomer NCO:OH 1:4 at different catalyst concentrations (200, 400, and 600 ppm) (Supplementary Fig. 14). **f** Evolution of G′ and G″ as a function of time at 37 and 60 °C for injectable tissue-mimetic elastomers prepared by reversible Diels-Alder crosslinking of furan (F) functionalized bottlebrushes with a linear dimaleimide (M) crosslinker at 1:1 molar ratio (F:M 1:1) (Supplementary Figs. 9 and 10).

Gelation is monitored by rheology, which identifies the crossover time between the storage (G′) and loss (G″) moduli at 37 °C (Fig. 1d). Within a given crosslinking scheme (e.g., NCO:OH), a combination of stoichiometry and temperature allows tuning gelation time (${t}_{{gel}}$) within more than two orders of magnitude as demonstrated by increasing ${t}_{{gel}}$ by simultaneously decreasing crosslinker concentration (Fig. 2a, b) and temperature (Fig. 2c, d), i.e., NCO:OH 1:1 at 50 °C (Fig. 2c) versus NCO:OH 1:8 at 20 °C (Fig. 2a). Similarly, switching from OH to NH_2_ functionalization decreases ${t}_{{gel}}$ from hours to minutes (Fig. 1d), which can be fine-tuned in the future by mixing OH- and NH_2_- terminated side chains into brushes. Overall, the injectable technology contains a toolbox of architectural and chemical parameters to enable broad tuning of cure time to cover a significant portion of biomedical applications. However, it is important to note that tuning crosslinker concentration inadvertently augments both the curing time and mechanical properties. To decouple ${t}_{{gel}}$ and *E*_0_ at a constant *T* = 37 °C, we prepared NCO:OH injectable formulations with different catalyst concentrations, which allows varying curing duration at a constant modulus of a fully cured elastomer (Fig. 2e). These decoupling efforts can be also explored through additional crosslinking chemistries (Fig. 1c) such as reversible Diels-Alder reactions which allow extending the curing time up to 11 h at 37 °C (Fig. 2f), which of particular interest to time-intensive body reconstructive procedures. In all of the studied systems, gel fraction ranges within 91–98%, which verifies the high efficiency of the developed solvent-free crosslinking schemes (Supplementary Tables 1, 2, 4).

### Tissue-mimetic mechanical properties

Mechanical properties of fully cured elastomers were evaluated by uniaxial tensile tests using the following equation of state relating true stress ${\sigma }_{{true}}$ with network elongation ratio $\lambda =L/{L}_{0}$ from its initial *L*_0_ to deformed size $L$:

1 $${\sigma }_{True}=\frac{E}{9}\left({\lambda }^{2}-{\lambda }^{-1}\right)\left[1+2\left(1-\frac{\beta ({\lambda }^{2}+2{\lambda }^{-1})}{3}\right)^{-2}\right]$$

This stress-elongation relationship has been validated for various synthetic and biological polymer networks^20,22,27^ and is described by two mechanical characteristics: Young’s modulus ${E}_{0}$ (Eq. 2) and firmness parameter $\beta$ (Eq. 3):

2 $${E}_{0}={\left.\frac{\partial {\sigma }_{{true}}}{\partial \lambda }\right|}_{\lambda \to 1}=\frac{E}{3}\left(1+\frac{2}{{\left(1-\beta \right)}^{2}}\right)$$

3 $$\beta =\left\langle {R}_{{in}}^{2}\right\rangle /{R}_{{\max }}^{2}$$

where $E$ is the structural modulus controlled by crosslink density. The firmness parameter 0 < *β* < 1 characterizes network’s strain-stiffening behavior described by extensibility of network strands from their initial end-to-end distance ${R}_{{in}}$ to the fully extended contour length ${R}_{{\max }}$. Therefore, the Young’s modulus ${E}_{0}$ of a polymer network depends not only on its crosslink density, but also on initial conformation of network strands ($\beta \sim \left\langle {R}_{{in}}^{2}\right\rangle$).

**Fig. 3:**
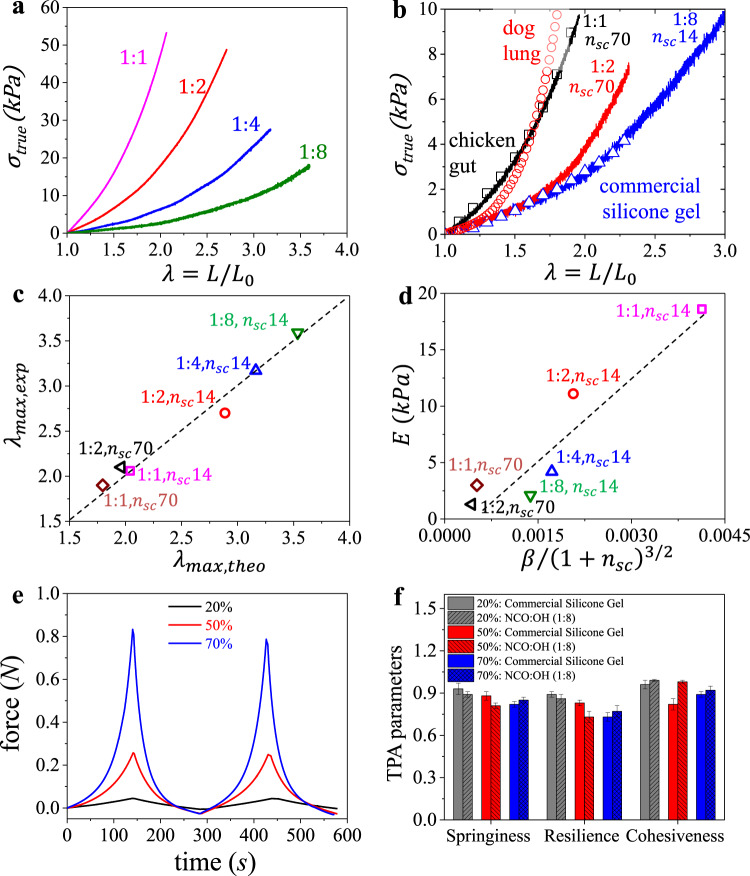
Replicating silicone gels mechanics with solvent-free injectable elastomers. **a** True stress vs. elongation curves of injectable elastomers prepared with different NCO:OH molar ratios. The decrease in crosslink density (1:1→1:8) results in concurrently decreasing softness (${E}_{0}$) and firmness (*β*). **b** Comparison of true stress-elongation curves of injectable elastomers with similar ${E}_{0}$ but different *β* relative to a commercial silicone gel with 70 wt% of sol fraction, and to different examples of weakly strain-stiffening biological tissues (e.g., chicken gut and dog lung)^27^. **c** Good agreement between experimental (${\lambda }_{{\max },{\exp }}$) and theoretical (${\lambda }_{{\max },{theo}}={\beta }^{-0.5}$) elongations-at-break suggests uniform mesh dimensions of injectable elastomers. **d** Linear correlation between the structural modulus ($E$) and ${\beta /(1+{n}_{{sc}})}^{3/2}$ validates architecturally tuning the mechanical properties of injectable brush elastomers. **e** Texture profile analysis (TPA) of the injectable elastomer NCO:OH 1:8 at different strain ratios of 20, 50, and 70%. **f** Comparing the TPA parameters (springiness, resilience, and cohesiveness) of the injectable elastomer NCO:OH 1:8 with the commercial silicone gel implant at different strain ratios of 20, 50, and 70%. At least *n* = ten independent TPA measurements of *k* = 3 independently prepared samples were conducted. Height of histogram bins and the error bars correspond to mean values ± standard deviation (SD), respectively.

For brush-like elastomers, these mechanical characteristics are controlled by three architectural parameters [${n}_{x},{n}_{{sc}},{n}_{g}$], which respectively correspond to the degree of polymerization (DP) of the bottlebrush backbone between two crosslinks, the side chains, or the backbone spacers between neighboring side chains. First, we explore ${n}_{x}$ by varying crosslinker concentration (NCO) at a constant molar fraction of OH-functionalized side chains (5 mol%). Similar to conventional linear chain polymer networks, increasing ${n}_{x}$ concurrently reduces the density of stress supporting strands and increases strand flexibility leading to enhanced softness at the expense of decreased firmness. This effect is clearly observed in Fig. 3a as decreasing the NCO:OH ratio (1:1→1:8) respectively results in lower Young’s modulus (${E}_{0}$) and less intense strain-stiffening, i.e., lower firmness ($\beta$). To increase firmness at a desired softness, we prepare bottlebrushes with longer side chains (${n}_{{sc}}=70$), which concurrently dilute the crosslinks and extend bottlebrush network strands. Respectively, bottlebrush elastomers with longer side chains maintain the gel-like softness, while enhancing network strain-stiffening toward tissue-relevant firmness (Fig. 3b). For instance, these injectable elastomers can closely match the softness and firmness of various weakly strain-stiffening biological tissues^27^ as shown in Fig. 3b. Importantly, all of the reported materials show excellent elasticity and demonstrate invariable and predictable stress–strain responses up until break (Supplementary Fig. 15).

Table 1 summarizes the corresponding ${E}_{0}$ and $\beta$ values extracted by fitting the stress–strain curves with Eq. 1. The obtained data highlight several important correlations. First, the brush network architecture allows independently tuning the softness (${E}_{0}$) and firmness ($\beta$) by concomitantly varying the [${n}_{x}$, ${n}_{{sc}}$] pair. For example, samples NCO:OH 1:4 with architectural pair [200,14] and NCO:OH 1:2 with [50,70] show significantly different firmness $\beta$ = 0.1 and 0.31 at a nearly constant Young’s modulus ${E}_{0}\cong\,$5 kPa. Second, the strand extensibility defined by a theoretical elongation-at-break ${\lambda }_{{\max },{theo}}={R}_{{\max }}/{R}_{{in}}={\beta }^{-0.5}$ (Eq. 3) demonstrates good agreement with the experimental ${\lambda }_{{\max },{ex}}$ of a macroscopic sample (Fig. 3c), which suggests a uniform network structure. Third, the structural Young’s modulus ($E$) of injectable elastomers is consistent with the theoretically predicted correlation $E\cong 3{k}_{b}T\frac{{l}^{3/2}}{{v}^{3/2}}\frac{\beta }{{\left(1+{n}_{{sc}}\right)}^{3/2}}$ (Fig. 3d), where ${k}_{b}$ – Boltzmann constant, $T$ – absolute temperature, $l$ – monomer length, and $v$ – monomer volume^23^, which reinforces the well-defined structure of injected polymer networks. Lastly, the mechanical properties are “invariant” with respect to crosslinking chemistry highlighting the flexibility of the injectable platform chemistry to only controlling curing duration and final product biocompatibility.

**Table 1:** Structural and mechanical parameters of various injectable formulations.

Crosslink chemistry^a^	Ratio^b^	${n}_{{sc}}$^c^	${n}_{{bb}}$^d^	${n}_{x}$^e^	*E* (kPa)^f^	*β*^g^	${E}_{0}$ (kPa)^h^	${\lambda }_{{\max }}^{{\exp }}$^i^	${\lambda }_{{\max }}^{{calc}}$ ^j^
Dual component permanent injectable elastomers									
NCO:OH	1:1	14	889	50	18.6	0.24	27.8	2.1	2.0
NCO:OH	1:2	14	889	100	11.1	0.12	13.5	2.7	2.9
NCO:OH	1:4	14	889	200	4.2	0.10	5.1	3.2	3.2
NCO:OH	1:8	14	889	400	2.1	0.08	2.3	3.6	3.5
NCO:OH	1:1	70	304	50	3.0	0.31	5.3	1.9	1.8
NCO:OH	1:2	70	304	100	1.3	0.26	2.1	2.2	2.0
Injectable elastomers with dynamic crosslinks									
F:M	1:1	14	889	50	15.3	0.23	22.3	2.1	2.1
F:M	2:1	14	889	100	6.3	0.14	7.8	2.7	2.6
F:M	4:1	14	889	200	1.5	0.12	1.8	2.9	2.8
Single component photocurable injectable elastomers									
PCMA	1.5	14	889	100	4.8	0.06	5.2	4.2	4.1
PCMA	3	14	889	200	1.7	0.05	1.8	4.9	4.5

^a^Chemistry used for injectable elastomers with isocyanate (NCO) coupled with hydroxyl (OH) or amine (NH_2_) (Figs. 1–3) furan (F) coupled with maleimide (M) (Supplementary Fig. 10) and photocurable methacrylate (PCMA) (Supplementary Fig. 11) side chain ends.

^b^Respectively ratios of each moiety in the chemistry couple. For PCMA, the ratio represents the controlled fraction percent of side chains with the photocurable moiety (i.e., 1.5 and 3 mol%).

^c^Degrees of polymerization (DP) of side-chains.

^d^Backbone of random polydimethylsiloxane-poly(ethylene glycol) (PDMS-*r*-PEG) bottlebrush macromolecules prior to crosslinking determined by ^1^H-NMR.

^e^Nominal DP of the backbone strand between cross-links.

^f^Structural Young’s modulus (*E*).

^g^Strain-stiffening parameter ($\beta$) obtained by fitting stress-strain curves with Eq. (1).

^h^Young’s modulus (Eq. 2).

^i^Experimental elongation at break.

^j^Theoretical elongation at break as ${\lambda }_{{\max },{theo}}={\beta }^{-0.5}$.

As mentioned above, injectable elastomer NCO:OH 1:8 with architectural parameters [400,14] demonstrate nearly identical softness and firmness with a silicone gel (~30 wt% gel fraction) extracted from a commercial breast implant (Fig. 3b), yet the solvent-free elastomers are more resilient and demonstrate significantly higher elastic deformation prior to fracture (${\lambda }_{{\max }}$). To further demonstrate the adequate mechanics of injectable elastomers, we conducted a texture profile analysis (TPA), whereby cylindrical samples are subjected to cyclic compressions at different deformations (Fig. 3e). From the TPA profiles, we evaluate several industrially relevant mechanical characteristics such as springiness, resilience, and cohesiveness (Supplementary Fig. 16) that favorably compare the solvent-free injectable elastomers with a commercial gel containing ~70% of liquid fraction (Fig. 3f).

### Non-leachability and cytocompatibility

**Fig. 4:**
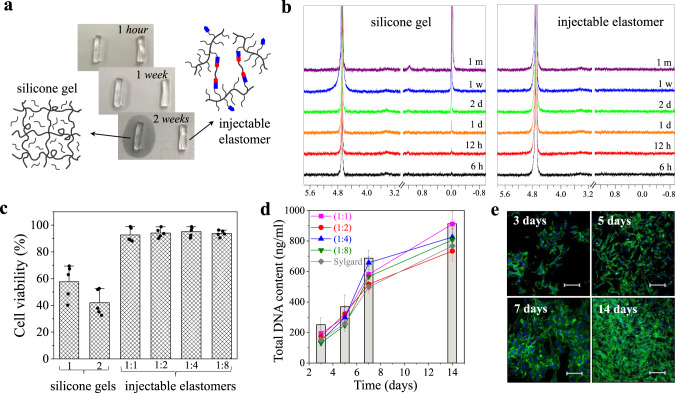
Leachability of gel-based implants and in vitro culture of cells on non-leachable injectable elastomers. **a** A paper-based test reveals leaching from a commercial silicone gel used in breast implants (Silicone Gel-1) versus non-leaching injectable silicone brush elastomer NCO:OH 1:8. **b** Time-resolved ^1^H-NMR of sol extract from the commercial silicone gel and a NCO:OH 1:8 injectable elastomer in D_2_O monitored over one month (400 MHz): 4.70 (residual H_2_O), 1.17, 0.01 (leachable materials). **c** Comparing cytotoxicity of commercial silicone gels and injectable silicone brush elastomers (NCO:OH 1:1→1:8) using NIH/3T3 fibroblasts. Dots in c depict individual samples. Height of the histogram bins and the error bars correspond to mean values ± SD, respectively. For the cytotoxicity test, 10^4^ cells/cm^2^ cells were examined over 5 independent experiments. **d** The extracted DNA quantification of cultured NIH/3T3 fibroblasts on injectable elastomers (NCO:OH 1:1→1:8), Sylgard (curing agent to base ratio of 1:10), and tissue culture polystyrene (bars) after 3, 5, 7, and 14 days. The data points and error bars correspond to mean values ± SD. For this test, 5 × 10^5^ cells/ml cells were examined over 5 independent experiments. **e** Proliferation of NIH/3T3 fibroblasts cultured to the injectable elastomer NCO:OH 1:8 monitored by fluorescence microscopy after 3, 5, 7, and 14 days (actin cytoskeleton and nucleus are displayed in green and blue, respectively). The scale bars correspond to 400 μm. The experiment was conducted on two independent cell lines in parallel showing similar results.

Gel-based implants, particularly organogels, perpetually leach various chemicals such as diluents, catalysts, and ligands into the body over time and upon deformation, which represents a significant long-term health concern^11,16,37^. This is readily observed by a commercially available silicone gel leaching onto a paper substrate (Fig. 4a), which is quantitatively corroborated by aqueous extraction of the sol fraction in time-resolved ^1^H-NMR (Fig. 4b) contrary to our non-leaching injectable elastomers. To further demonstrate the significance of leachable-free compositions, we compare cytotoxicity^38^ and cell proliferation between gels and injectable elastomers^39,40^. Cytotoxicity tests are performed according to ISO 10993-5 for the aqueous extractions (Fig. 4c) with a NIH/3T3 fibroblast viability above 90% when exposed to extracts from the injectable formulations after 24 h (Fig. 4c), while extracts from commercial silicone gel implants show significantly diminished viability of 40–60%. Further, the proliferation of NIH/3T3 fibroblasts is analyzed by measuring the total DNA content of cultured fibroblasts. The total extracted DNA from cultured cells on elastomer surfaces confirms increasing cell count over two weeks for each injectable formulation (Fig. 4d). Minimal deviation between fibroblast proliferation rate on injectable elastomers versus the tissue culture polystyrene (TCPS) as reference implies the elastomer samples do not stimulate fibroblast proliferation as visually demonstrated by time-resolved fluorescence imaging (Fig. 4e), which affirms the use of injectable elastomers as biocompatible materials. This neutral response may be explained by both the injectable elastomers hydrophobic nature precluding cell interaction within the material bulk and the minimal tissue exposure to surficial functional groups. For instance, a 1 mL injection of a NCO:OH 1:1 with architectural pair [50,14] contains a surface density of reactive groups of ~5 × 10^−10^ mm^−2^, which is much smaller than 1 ppb and is further reduced for NCO:OH 1:1→1:8.

### In vivo administration of injectable elastomers

**Fig. 5:**
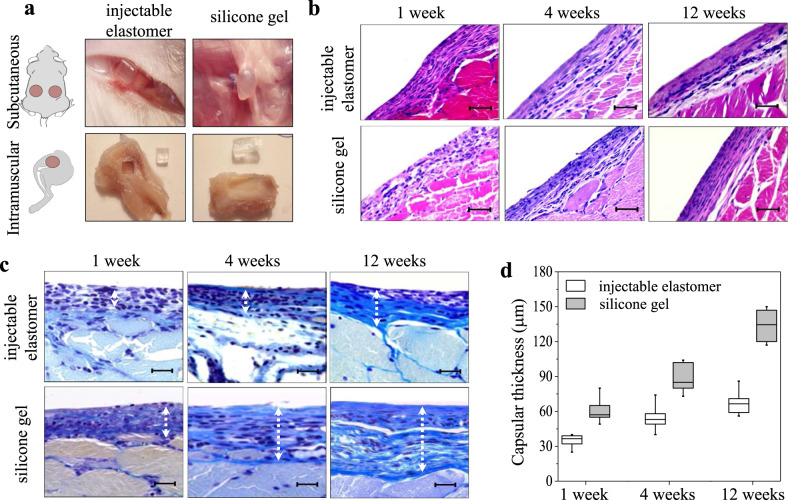
Characterization of injectable elastomers in vivo. **a** Schematic and explanted specimens of the injectable elastomer NCO:OH 1:8 (left), and Silicone Gel-1 (right) after 12 weeks subcutaneous (top), and intramuscular (bottom) administration. The ex vivo cured implanted elastomer maintains its implanted rectangular shape with sharp edges, while the silicone gel becomes round and exhibits traces of partial fragmentation. **b** Histology of intramuscular specimens at 1, 4, and 12 weeks explanation of the injectable elastomer NCO:OH 1:8 and Silicone Gel-1 stained with hematoxylin and eosin, and **c** using the Mallory’s procedure. The scale bars in **b**, **c** correspond to 100 and 50 μm, respectively. **d** Comparing thickness of the fibrous layer in injectable elastomer NCO:OH 1:8 and Silicone Gel-1 explanted at 1, 4, and 12 weeks. The capsular thickness was measured in 10 different locations for *n* = 6 male Wistar white rats at 1, 4, and 12 weeks. The boxplot displays the distribution of the raw data. For each box, the middle line corresponds to the median, while the lower and upper hinges correspond to the first and third quartiles, the upper whisker extends from the hinge to the largest value no further than 1.5 × IQR from the hinge (where IQR is the inter-quartile range) and the lower whisker extends from the hinge to the smallest value at most 1.5 × IQR of the hinge.

Traditionally, encapsulation of silicone gels within a stiff impermeable shell has been employed to control leaching rate^11^. However, many reports show limited improvement as the shell material is permeable to small molecules^9^ and is significantly stiffer than surrounding tissue instigating capsular growth^7^. Therefore, we conduct in vivo assessment of our injectable elastomers using animal models subjected to both subcutaneous and intramuscular implantation. To ensure a fair comparison with a commercial silicone gel, ex vivo cured elastomer samples are implanted through an incision similar to reconstructive tissue implants (Fig. 5). In addition, we inject one of our formulations directly in vivo to evaluate the compatibility of the in situ curing process (Supplementary Fig. 17). The injected samples are found to be localized at the injection site with no visible dispersion into the surrounding tissue. In each case, explanted samples are well tolerated, with no clinical evidence of inflammatory response in surrounding tissues (Fig. 5a). In the subcutaneous explants, a thin translucent layer of encapsulating connective tissue is observed, which is significantly thicker around silicone gels. In muscle tissue, the injectable samples are fully intact and can be thoroughly explanted in contrast to the disfigured and partially fragmented silicone gels.

According to the Hematoxylin-Eosin and Mallory Trichrome staining overview, the injectable elastomer capsule does not contain multinucleated foreign body giant cells at any stage and does not contain lymphocytes, leukocytes, macrophages on later stages, suggesting the implanted materials preclude chronic inflammation and are sufficiently inert (Fig. 5b, c). The capsular thickness of the fibrous layer was quantified by morphometric image analysis on the Mallory’s trichrome stained slides (Fig. 5c). The ex vivo cured implanted elastomers display significantly lower capsular thickness compared to silicone gels at 1, 4, and 12 weeks (Fig. 5d), which may be ascribed to both the lack of leaching into the animal body and their tissue-matching softness. For directly injected samples, the histology analysis shows transient inflammatory response for 7, and 14 days (Supplementary Fig. 17).

To assess injectable elastomers long-term stability under physiological conditions, their mechanical properties were re-evaluated after (i) implantation in vivo (Supplementary Fig. 18), and (ii) incubation in phosphate-buffered saline, pH 7.4 at elevated temperature 70 °C (Supplementary Fig. 19). Both in vivo and ex vivo tests did not reveal any significant deviation in deformation response. It is important to note that the hydrophobic nature of the injectable elastomers inhibits water permeability, which excludes hydrolytic degradation of the network.

It should be noted that in vivo spreading of an injectable formulation might not be desired for some applications. However, this is a nonissue for the fast-curing injectable elastomer system (e.g., NCO: NH_2_), while the slowly curing systems may circumvent this problem through small injections of the highly viscous and hydrophobic materials as demonstrated through injectable implantation (Supplementary Fig. 17). For high-volume injection applications (e.g., breast implants), traditional lumens may be used as mentioned above.

## Discussion

Soft tissues demonstrate a very distinct response to deformation: they are soft when touched, yet rapidly stiffen upon deformation. Synthetic replication and in vivo implementation of this duality is imperative for body fillers and reconstruction of various tissues after disease and injuries including HIV-associated lipoatrophy, mastectomy or lumpectomy, burns, tumor removal, and general bodily injuries. These traumas not only represent physical concerns such as severe sitting pain after loss of adipose tissue in the buttocks^41^, but also psychological stresses after mastectomy^42,43^ and discrimination after loss of tissue in the face^44,45^. Although current implant strategies attempt to address these issues, they also introduce a host of additional undesired consequences. For instance, current devices only replicate tissue softness by employing various kinds of gels, i.e., by diluting polymer networks with liquids, which continually leach into the body as commonly experienced by women who undergo breast reconstruction with silicone (0.06 mg/liter) detected in their breast milk^46^. Furthermore, implant’s significant firmness mismatch with surrounding tissue creates additional health risks and psychological issues due to capsular contracture and disfigurement, which requires future invasive explanation surgeries. To mitigate surgical complications, different injectable technologies have been introduced such as injecting polyacrylamide microgels (PAAG). Although initially thought to be safe, PAAGs are now banned in most countries due to substantial evidence associating PAAG with infection, glandular atrophy, fibrosis, inflammation, and palpable scleroma formation after water fraction absorption of migrated microgel fragments throughout the body^9,47,48^. Given these outlined circumstances, we believe that our minimally invasive injectable, non-leaching, tissue-mimetic, and biocompatible elastomer platform will advance various biomedical device applications. Architecturally tailored brush-based mesoblocks augmented with functionalized side chains enable both tunable curing time and tissue-like mechanical properties of fully cured materials. The formulations contain no solvent and do not leach, while bottlebrush architecture of chains enables injectability due to significantly lower brush melt viscosity compared to linear chains of similar molecular weight. These features coordinate to bestow sufficient biocompatibility as demonstrated both in vitro and in vivo. Although this tissue-mimetic (Fig. 3b) technology promises to revolutionize reconstructive implantation procedures, future work will be aimed to expand the crosslinking schemes and architectural landscape to independently control curing duration with softness and firmness combinations that mimic any tissue. The design-by-architecture approach is adaptable to any chemistry, which will allow future expansion of this platform to other commodity polymers such as polyolefins, polyacrylates, and polyesters. Furthermore, side-chain functionalization opens many opportunities for precision engineering of alternative applications such as tissue adhesives, and coating of implanted medical devices to enhance biocompatibility and performance^40^. Last but not least, the devised injectable platform is readily applicable to fabricate soft medical implants with tissue-mimetic mechanics via additive manufacturing techniques^29^.

## Methods

### Materials

Monomethacryloxypropyl-terminated polydimethylsiloxane macromonomers (MCR-M11: *M*_*n*_ ~ 1000 g/*mol*, ${n}_{{sc}}$14, and MCR-M17: *M*_*n*_ ~ 5000 g/*mol*, ${n}_{{sc}}$70) were obtained from Gelest and purified using basic alumina columns to remove inhibitor. Aminopropyl terminated polydimethylsiloxane (DMS-A15, M_*n*_~3,000 g/*mol*), trimethylsiloxy terminated polydimethylsiloxane (DMS-T72, *M*_*n*_ ~ 500,000 g/mol), and chlorine terminated polydimethylsiloxane (DMS-K05, *M*_*n*_ ~ 425–650 g/mol) were purchased from Gelest and used as received. PEGMA macromonomer (*M*_*n*_ ~ 500 g/mol) was obtained from Sigma-Aldrich and purified using basic alumina columns to remove inhibitor. Ethylene bis(2-bromoisobutyrate) (2f-BiB), tris[2-(dimethylamino)ethyl]amine (Me6TREN), Copper(I) chloride (CuCl), Copper(I) bromide (CuBr), triethylamine (TEA), methanesulfonyl chloride, tris(hydroxypropyl)phosphine (THPP), Sodium azide, isophorone diisocyanate (IPDI), and Tin(II) 2-ethylhexanoate, furfuryl isocyanate, maleic anhydride, furan, ethanolamine, isocyanatoethyl methacrylate were purchased from Sigma-Aldrich and used as received. Toluene, anisole, isopropanol, dichloromethane (DCM), *N,N*-dimethylformamide (DMF), and tetrahydrofuran (THF) were purchased from VWR Chemicals and used as received.

### Synthesis of brush polymers

To design injectable tissue-mimetic elastomers, polydimethylsiloxane (PDMS) brushes with a predetermined fraction of functionalizable end-groups on the side-chains were synthesized through controlled radical copolymerization of PDMSMA and PEGMA macromonomers. A detailed procedure of atom transfer radical polymerization of random polydimethylsiloxane-poly(ethylene glycol) brushes (PDMS-*r*-PEG) is as follows: A 250 ml Schlenk flask equipped with a magnetic stir bar was charged with 16.0 mg 2f-BiB, 50.0 g MCR-M11, 20.5 mg Me6TREN, 1.25 g PEGMA (5 mol%), and 100 ml toluene. Prior to reaction, the solution was bubbled with dry nitrogen for 1.5 h, then 8.8 mg Cu(I)Cl was rapidly added to the reaction mixture under nitrogen atmosphere. The flask was sealed, purged for 15 min, and then immersed in a 45 °C oil bath. The polymerization was stopped after 4 h to yield 80% macromonomers conversion as verified by ^1^H-NMR, resulting in a PDMS-*r*-PEG brush polymer with degree of polymerization (DP) of the backbone ~900. The polymer was precipitated three times in DMF to purify residual macromonomers. The resulting purified polymer was dried under vacuum at room temperature until a constant mass was reached. ^1^H-NMR spectra of PDMS-*r*-PEG brushes at different time points are illustrated in Supplementary Fig. 2. The growth kinetics of PDMS-*r*-PEG shown in Supplementary Fig. 3 confirms a random distribution of macromonomers in the brush backbone. ^1^H-NMR of PDMS-*r*-PEG at different stages of synthesis has been shown in Supplementary Fig. 4.

In order to synthesize PDMS-*r*-PEG with long side-chains (${n}_{{sc}}$70), a 100 mL Schlenk flask was equipped with a stir bar and charged with 9.6 mg 2f-BiB, 50 g MCR-M17, 250 mg PEGMA, 12.2 mg Me_6_TREN and a solvent mixture of anisole (40 ml) and toluene (10 ml). The solution was bubbled with dry nitrogen for 1.5 h, then 7.6 mg Cu(I)Br was rapidly added to the reaction mixture under nitrogen atmosphere. The flask was sealed, purged for an additional 15 min, and then immersed in a 45 °C oil bath. The polymerization was stopped after 5 h to yield 80% monomer conversion as verified by ^1^H-NMR (Supplementary Fig. 5), resulting in a PDMS-*r*-PEG brush polymer with DP of the backbone ~300. The polymer was precipitated three times from isopropanol to purify residual macromonomers. The resultant purified polymer was dried under vacuum at room temperature until a constant mass was reached.

### Synthesis of azide-terminated macromonomer

The following procedure was performed to synthesize amine-terminated PEGMA macromonomer. A 100 ml round-bottom flask equipped with a magnetic stir bar was charged with 10 g PEGMA, 50 ml DCM, and 2.5 g TEA, sealed and then placed in an ice bath. Subsequently, 2.5 g methanesulfonyl chloride was added drop-wise to the mixture using a syringe pump, and reaction was stirred overnight. The resultant solution was passed through column for purification, and then dried. The obtained PEG derivate along with 50 ml DMF and 3 g sodium azide were charged into a 100 ml round-bottom flask equipped with a magnetic stir bar. The reaction was stirred for 24 h at room temperature. The mixture was centrifuged to remove excess salt, dried, and then azide-terminated PEGMA macromonomer was extracted by dissolving in DCM followed by washing with water. ^1^H-NMR spectrums of PEG macromonomer functionalization at different stages are shown in Supplementary Fig. 6.

### Synthesis of amine-functionalized brushes

A similar method as described above was followed to synthesize brush polymers using the PDMSMA and azide-terminated PEGMA macromonomers. After purification of the brushes, they were dissolved in anhydrous THF, reacted with excess THPP for 24 h, and then water was added to the mixture. Finally, the amine-functionalized brushes were purified via passing through column, and then dried for further use. ^1^H-NMR spectra of PDMS-*r*-PEG.N_3_ and PDMS-*r*-PEG.NH_2_ brush copolymers are displayed in Supplementary Fig. 7.

### Synthesis of polydimethylsiloxane diisocyanate crosslinker

In order to synthesize PDMS macromolecular crosslinker, a 100 ml round-bottom flask equipped with a magnetic stir bar was charged with 10 g IPDI, 50 ml anhydrous DCM, and sealed. Afterward, 5 g DMS-A15 dissolved in 10 ml anhydrous DCM was added drop-wise to the mixture using a syringe pump. Subsequently, the resulting PDMS diisocyanate crosslinker was purified to remove excess IPDI and dried for further use. Supplementary Fig. 8 displays the ^1^H-NMR of PDMS diisocyanate crosslinker at different stages of synthesis.

### Preparation of injectable tissue-mimetic elastomers

Two sets of complementary chemistry were used to prepare injectable tissue-mimetic elastomers from mixtures of functionalized brushes and crosslinkers: isocyanate:hydroxyl (NCO:OH) and isocyanate:amine (NCO:NH_2_). In the former case, PDMS-*r*-PEG brushes were mixed with predetermined amount of PDMS diisocyanate crosslinker to reach different NCO:OH molar ratios of 1 to 1, 2, 4, and 8, in the presence of 100 ppm Tin(II) 2-ethylhexanoate, and then cured. In the case of NCO:NH_2_ injectable elastomer, PDMS-*r*-PEG brushes were mixed with predetermined amount of PDMS diisocyanate crosslinker to reach predetermined crosslink density. Supplementary Fig. 20 and Supplementary Video 1 demonstrate administration and handling of an injectable elastomer by means of a double syringe system.

### Synthesis of injectable dynamic tissue-mimetic elastomers

Reversible Diels-Alder chemistry was used to prepare injectable dynamic tissue-mimetic elastomers from mixtures of functionalized brushes and a difunctional crosslinker (Supplementary Figs. 9, 10). To substitute hydroxyl end groups with diene moieties, hydroxyl-functionalized bottlebrushes (PDMS-*r*-PEG) were reacted with excess furfuryl isocyanate in the presence of DBTDL as catalyst in anhydrous dichloromethane. In order to synthesize linear bifunctional dienophile PDMS crosslinker, *exo*-3,6-epoxy-1,2,3,6-tetrahydrophthalic anhydride (furan-protected maleic anhydride) was first synthesized. In brief, A 500 ml round-bottom flask equipped with a magnetic stir bar was charged with 50 g maleic anhydride and 250 ml toluene. The mixture was heated to 80 °C, and subsequently, 55.6 ml furan was added. The mixture in the capped flask was cooled to room temperature, and the reaction proceeded 24 h at room temperature. The resultant precipitate was filtered, washed with diethyl ether, and dried. In the next step, 25 g of the synthesized furan-protected maleic anhydride was charged into a 500 ml round-bottom flask equipped with a magnetic stir bar and dissolved in 100 ml methanol. The flask was sealed, purged for 15 min, and then immersed in an ice bath. To this solution, ethanolamine (17 mL, 17.2 g, 0.281 mol) was added via syringe. The reaction mixture was stirred at 0 °C for 30 min, and then refluxed for14 h. After reacting, the solution was cooled to room temperature, and then cooled to −20 °C. The product, furan-protected N-(2-hydroxyethyl) maleimide (Fp-HEMI), crystalized out of solution at −20 °C. The solid was collected by filtration and washed with isopropanol and allowed to dry. Afterward, chlorine terminated PDMS was reacted with Fp-HEMI, refluxed, and purified to achieve maleimide terminated PDMS as linear bifunctional crosslinker for injectable dynamic tissue-mimetic elastomers (Supplementary Fig. 9).

### Synthesis of injectable photocurable tissue-mimetic elastomers

To substitute hydroxyl groups on PDMS-*r*-PEG with photocurable methacrylate moieties, bottlebrushes comprising different molar ratio of hydroxyl side-chains end groups were reacted with excess 2-isocyanatoethyl methacrylate in the presence DBTDL as catalyst in anhydrous dichloromethane (Supplementary Fig. 11). Subsequently, the functionalized bottlebrushes were precipitated two times in anhydrous dimethylformamide to purify residual IEM and DBTDL. Finally, the functional bottlebrushes were dried with dry N_2_ flow until a constant mass was reached. The functionalized brushes were subsequently cured in the presence of diphenyl(2,4,6-trimethylbenzoyl)phosphine oxide/2-hydroxy-2-methylpropiophenone as photo-initiator under N_2_ using a UV illumination chamber (365 nm UV lamp, 0.1 mW/cm^−2^, 10 cm distance).

### Rheological measurements

Evolution of elastic (G′) and loss (G″) moduli as a function of time for injectable formulations at different temperatures and compositions were measured at angular frequency of 1 rad s^−1^ and oscillation strain of 0.5% using an ARES-G2 rheometer from TA Instruments. In order to evaluate stability of the premixed injectable formulations at low temperature, their rheological properties were monitored overtime at 0 °C. The formulations remain fluid, which demonstrate the feasibility of their long-term storage at low temperature (Supplementary Fig. 21). PDMS-*r*-PEG bottlebrush melts as the precursor of injectable elastomers were studied for their rheological properties in terms of viscosity as a function of shear rate, complex viscosity as a function of oscillation strain, G′ and G″ moduli as a function of oscillation strain at 25 and 37 °C (Supplementary Fig. 22). Further, cured injectable elastomers (NCO:OH) were characterized for their viscoelasticity as a function of frequency at 37 °C (Supplementary Fig. 23).

### Injectability measurements

Injectability of PDMS-*r*-PEG bottlebrush melts as the precursor of injectable elastomer formulations was measured by means of a desktop bioprinter BIO X (CELLINK) with piston‐driven syringe heads and pneumatic printheads. Mass flow rate of bottlebrush melts was monitored as a function of injection pressure using different nozzles (16G and 20G) at 25 and 37 °C (Supplementary Fig. 24, Supplementary Video 2).

### Uniaxial tensile stress–strain measurements

Samples were cut into dogbone shape with bridge dimensions of 12 mm × 2 mm × 1 mm, loaded to a RSA-G2 DMA (TA Instruments), and subjected to uniaxial extension at 20 °C and constant strain rate of 0.005 s^−1^. Samples were stretched until rupture to determine the entire mechanical profile. For each sample, tests were conducted in triplicate to ensure accuracy of the data. All stress-strain curves show dependence of the true stress, ${\sigma }_{{true}}$, on the elongation ratio $\lambda =L/{L}_{0}$ in accordance with Eq. 1. The elongation ratio $\lambda$ for uniaxial network deformation is defined as the ratio of the sample’s instantaneous size $L$ to its initial size ${L}_{0}$, $\lambda =L/{L}_{0}$. The Structural and mechanical parameters of reported injectable elastomers are summarized in Table 1. Moreover, to assess long-term stability of the injectable elastomers under physiological conditions, their mechanical properties were re-evaluated after incubation in phosphate buffered saline, pH 7.4 at elevated temperature of 70 °C (Supplementary Figs. 18 and 19).

### Texture profile analysis

In order to examine how injectable elastomers behave when deformed, texture profile analysis (TPA) was performed using an RSA-G2 DMA (TA Instruments) in compression mode. Disk-shaped samples with 8 mm diameter were compressed twice, and their behavior at different strain ratios of 20, 50, and 70% was monitored. TPA parameters (springiness, resilience, and cohesiveness) were measured based on force–time curves. In addition, in order to evaluate long-term stability of the injectable elastomers under physiological conditions, their texture profile was re-analyzed after implantation in vivo (Supplementary Fig. 18).

### Elastomers bleed (leachability) tests

In order to monitor the leachability of injectable elastomers in comparison with silicone gels used in commercial breast implants, elastomers were immersed in an aqueous medium and the leached residues were monitored using ^1^H-NMR at different time intervals over one month (Supplementary Fig. 25). In order to quantify the leachable fraction from three types of commercial silicone gels and our injectable elastomer after one month, the leached residues in an aqueous medium were freeze-dried, and their mass was measured, and reported based on sample weight (Supplementary Fig. 26). Furthermore, to visualize the leachable diluent fraction from a commercial silicone gel in comparison with the injectable elastomers, bulk samples were placed on a paper substrate and monitored over time (Supplementary Fig. 27).

### Atomic force microscopy

Atomic force microscopy (AFM) was performed in PeakForce QNM mode using a multimode AFM (Brüker) with a NanoScope V controller and silicon probes (resonance frequency of 50–90 Hz and spring constant of ~0.4 N/m). Based on obtained height micrographs of PDMS-*r*-PEG brushes deposited on mica by Langmuir-Blodget technique for ${n}_{{sc}}$14 and ${n}_{{sc}}$70 brushes ${n}_{{bb}}$ was determined as ${L}_{n}/{l}_{0}$, where ${L}_{n}$ is number average measured brush contour length via AFM, and ${l}_{o}$= 0.25 nm is the length of brush backbone monomeric unit. Brush chains dispersity, *D* = *M*_w_/*M*_n_ was calculated from analysis of >300 molecules. The dimensions of brush chains were extracted from AFM images in Supplementary Fig. 12, and were found to be consistent with expected dimensions determined by ^1^H-NMR in Supplementary Table 3, and gel permeation chromatography (Supplementary Fig. 28).

### Gel permeation chromatography

GPC was performed on a Waters 2695 separations module liquid chromatograph equipped with either four Waters Styragel HR columns arranged in series or two Agilent Resipore columns maintained at 35 °C, and a Waters 2414 refractive index detector at room temperature. THF was used as the mobile phase at a flow rate of 1.0 mL/min. Molecular weight and dispersity data were reported relative to polystyrene standards.

### Cytotoxicity assay

According to ISO standard 10993-5, the cytotoxicity of extracts from the injectable elastomers through direct injection and commercial silicone gels was studied to resemble the cellular behaviors in the first 24 h of the clinical applications. Samples were prepared according to the ISO requirements, by placing them into the extraction medium containing Dulbecco’s modified Eagle’s medium (DMEM), with 10% fetal calf serum (FCS) and 1% mixture of Penicillin/Streptomycin (Sigma-Aldrich) at a concentration of 3 cm^2^/mL at 37 °C and 5% CO_2_ for 24 h. Two types of cells including fibroblasts (NIH/3T3) and human umbilical vein endothelial cells (HUVECs) both from American Type Culture Collection were used. The cells were seeded on 96-well plate at an initial concentration of 10^4^ cells/cm^2^. After 24 h incubation, the extracts were added to the cells, and the cell viability was measured after 24 h by resazurin-based PrestoBlue cell viability reagent (Invitrogen) according to the manufacturer’s instructions. At the end of incubation time, the culture medium was replaced with measurement solution containing 10% of PrestoBlue reagent, and the fluorescence intensity was measured using a microplate reader (Biotek Instruments) at excitation wavelength of 544 nm, and an emission wavelength of 590 nm, after 30 min of incubation. Supplementary Fig. 29 shows viability data for HUVECs, which was found to be consistent with the results obtained using NIH/3T3 cells.

### Cell proliferation assay

NIH/3T3 proliferation assay in contact with injectable elastomers was performed over 2 weeks. Silicone elastomer (curing agent to base ratio of 1:10, Sylgard 184, Dow Corning) and TCPS were studied as control. To quantify the cellular proliferation, cells were seeded at the density of 5 × 10^5^ cells/ml in a 12-well plate. The culture medium was containing DMEM basal media supplemented with 10% FCS and 1% Penicillin/Streptomycin. After 24 h incubation, the injectable formulations were injected directly into the culture medium. At 3, 5, 7, and 14 days, the DNA content of the cells was quantified via the Quanti-iT PicoGreen dsDNA kit (Invitrogen) based on the manufacturer’s instructions. Further, immunohistochemical staining was performed to monitor the cell number using Cytopainter Green Fluorescence F-actin staining kit, and the 4′,6-diamidino-2-phenylindole following the manufacturer’s instructions.

### Animal Study

For the in vivo analysis, male Wistar white rats with age: 6–7-week-old (350–400 g) were used in accordance with principles of the European Convention, Strasbourg, 1986 and the Helsinki Declaration of the World Medical Association for the Humane Treatment of Animals 1996. The animals were kept on a 12/12 h light/dark cycle, in a temperature and humidity-controlled environment with food and water made available ad libitum. The surgical implantations were carried out under an aseptic condition. To ensure direct comparison and eliminate the effects of the surgical administration procedure, under general anesthesia, the annealed injectable elastomers and commercial gel samples with nearly identical mechanical properties (Fig. 3b) were implanted in the musculus adductor magnus on both hind limbs and subcutaneous. Six rats (*n* = 6) were used in each group with two transplants in each. After operation, rats were housed in individual cages. The implanted samples were harvested at 1, 4-, and 12-weeks post-surgery. For histological tissue analysis, the explanted samples were fixed in 10% neutral formalin in phosphate buffer (pH 7.4) for 72 h and then dehydrated in a series of ethanol solutions (50%, 70%, 90%, and 100% ethanol, 5 min each) and embedded in paraffin. The 5 µm paraffine slides were sectioned using a microtome. The slides were then stained with hematoxylin and eosin (H&E) after deparaffinization and rehydrated in a graded ethanol solution series (100%, 90%, and 70% ethanol, 5 min each; dH_2_O for 10 min). For visualization of connective tissue, the Mallory’s trichrome (Bio-Optica) was used. Further, microscopic analysis was carried out using a Leica DM750 light microscope (Leica), and digital images were recorded using an ICC 50 camera (Leica).

### Reporting summary

Further information on research design is available in the Nature Research Reporting Summary linked to this article.

## Supplementary information

Supplementary Information

Description of Additional Supplementary Files

Supplementary Video 1

Supplementary Video 2

Reporting Summary

## Data Availability

The authors declare that all data supporting the findings of this study are available within the paper and its supplementary information files. The latter includes full characterization of synthesis and mechanical testing of injectable elastomers reported herein. The respective raw data are available from the corresponding author upon reasonable request.
