# Supplementary material for: Injectable non-leaching tissue-mimetic bottlebrush elastomers as an advanced platform for reconstructive surgery
Source: Nat Commun. 2021 Jun 25;12:3961. doi: 10.1038/s41467-021-23962-8 (PMC8233362; doi:10.1038/s41467-021-23962-8)
Supplement: Supplementary file 1 — Supplementary Information [file 41467_2021_23962_MOESM1_ESM.pdf]

Supplementary Information

Injectable Non-leaching Tissue-mimetic Bottlebrush Elastomers as an Advanced Platform for Reconstructive Surgery

Erfan Dashtimoghadam[1], Farahnaz Fahimipour[1,2], Andrew N. Keith[1], Foad Vashahi[1], Pavel Popryadukhin[3], Mohammad Vatankhah-Varnosfaderani[1*], Sergei S. Sheiko[1*]

[1]*Department of Chemistry, University of North Carolina at Chapel Hill, 27599, USA*

[2]*Division of Comprehensive Oral Health, Periodontology, Adams School of Dentistry, University of North Carolina at Chapel Hill, Chapel Hill, NC, USA*

[3]*Instituteof Macromolecular Compounds of the Russian Academy of Sciences, St.Petersburg, Russia*

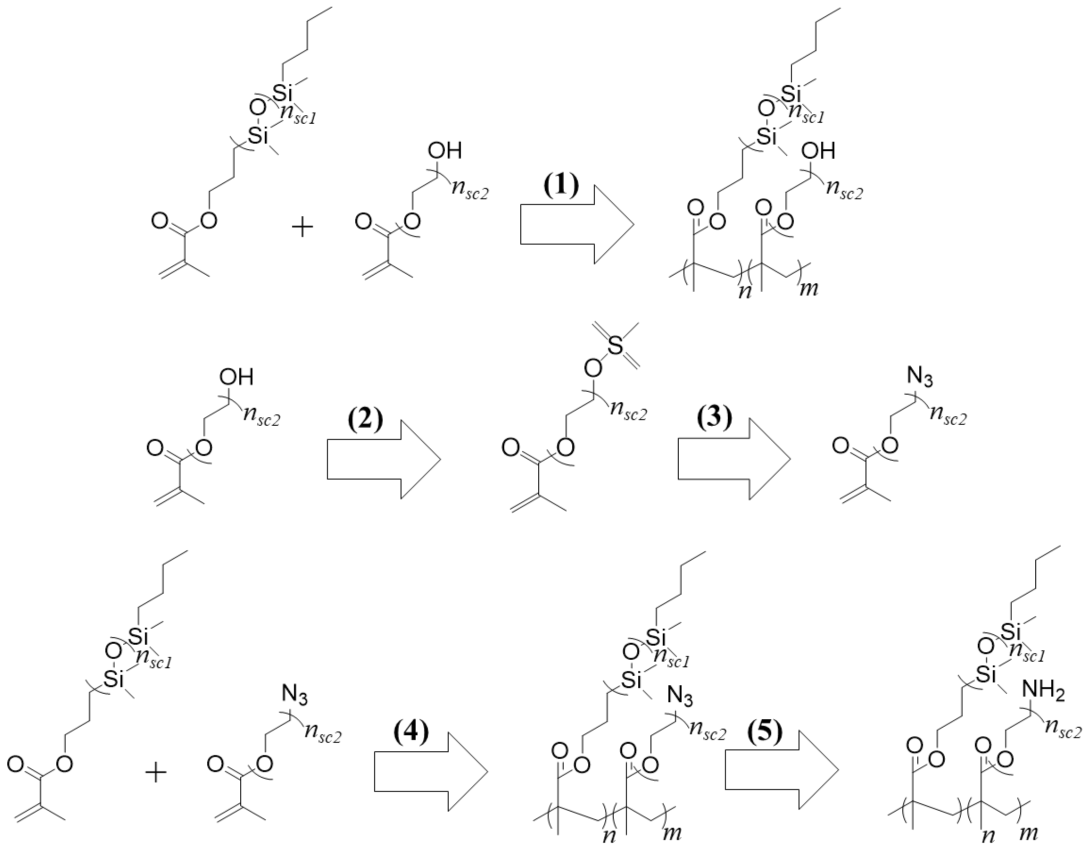

Figure 1. Synthesis of functional bottlebrush polymers and macromonomers: **(1)** synthesis of random polydimethylsiloxane-poly(ethylene glycol) bottlebrush copolymer (PDMS-*r*-PEG) through controlled radical copolymerization of polydimethylsiloxane-methacrylate (PDMSMA) and polyethyleneglycol-methacrylate (PEGMA) macromonomers, **(2)** mesylation of PEGMA macromonomer, **(3)** synthesis of azide-terminated PEGMA from mesylated macromonomer, **(4)** synthesis of random polydimethylsiloxane/azide-terminated poly(ethylene glycol) (PDMS-*r*-PEG.N_3) bottlebrush copolymer, and **(5)** reduction of PDMS-*r*-PEG.N_3 to achieve PDMS-*r*-PEG.NH_2 bottlebrush copolymer (for details of illustrated reactions, please see Methods Section).

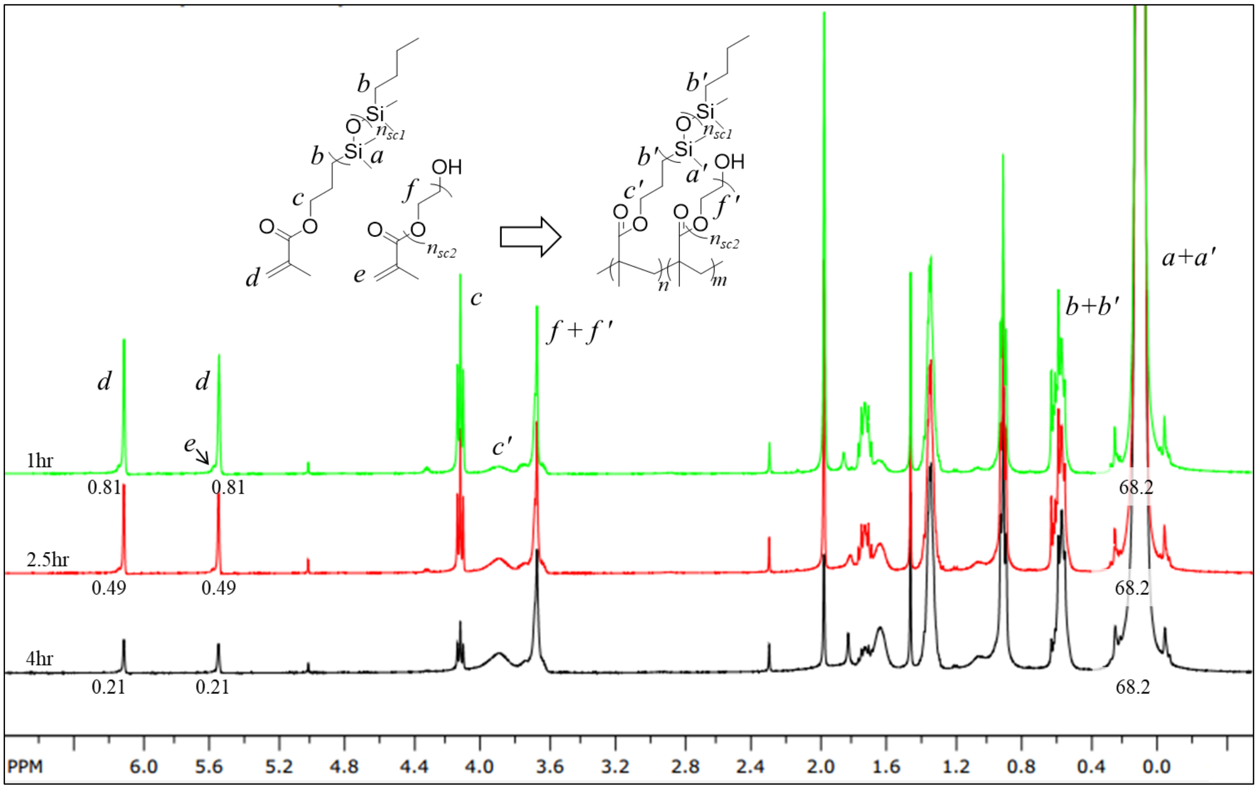

Figure 2. ^{1}H-NMR growth of a random polydimethylsiloxane-poly(ethylene glycol) brush (PDMS-*r*-PEG, *n*:*m*, 95:5, n_{sc1}: 14, n_{sc2}: 12) (400 MHz, $CDCl_3$): 6.16, 5.57 ($C\mathbf{H_2}$=C(CH_3)C=O, PDMS and PEG macromonomer mixture, s, 1H), 4.12 (CO-OC$\mathbf{H_2}$-, PDMS macromonomer, t, 2H), 3.91 (CO-OC$\mathbf{H_2}$-, PDMS brush, t, 2H), 3.78 (CO-OC$\mathbf{H_2}$-, PEG brush, t, 2H), 3.67 (-$OC_2\mathbf{H_4}O$-, PEG brush, m, 32H), 0.55 (-CH_2-(Si$(CH_3)_2$-O$)_n$-C$\mathbf{H_2}$-CH_2-, PDMS macromonomer and brush mixture, m, 4H), 0.09 (-(Si(C$\mathbf{H_3})_2$-O$)_n$-, PDMS macromonomer and brush mixture, s, 68.2H). $Conv_{PDMS} = ([Area(a+a')/68.2] \; - [Area(d)/1])/[Area(a+a')/68.2]=$ 79%. $n_{bb} = Conv_{PDMS} * \frac{[M]}{[I]} = 79\% * 1125 = 889.$

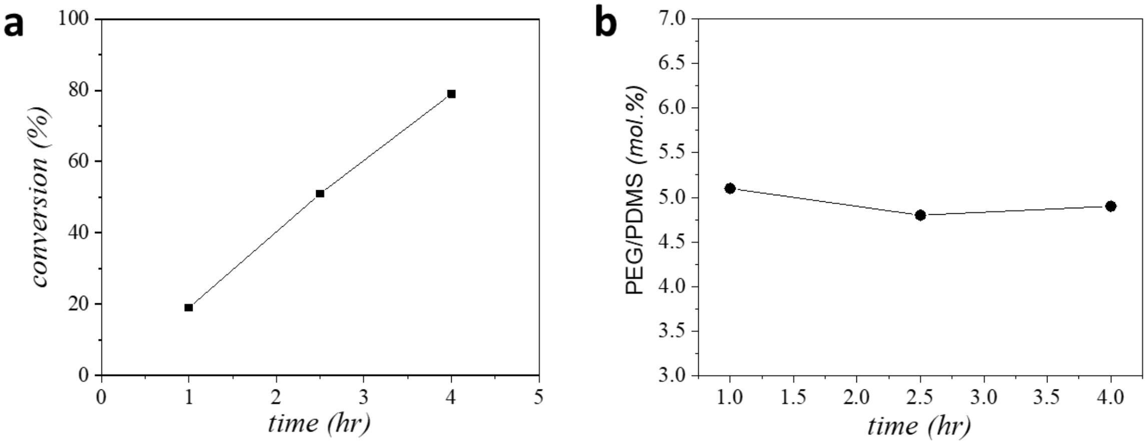

Figure 3. **a**, Growth kinetics of random polydimethylsiloxane-poly(ethylene glycol) (PDMS-*r*-PEG) copolymer bottlebrushes. **b**, Molar ratio of PEG during copolymerization.

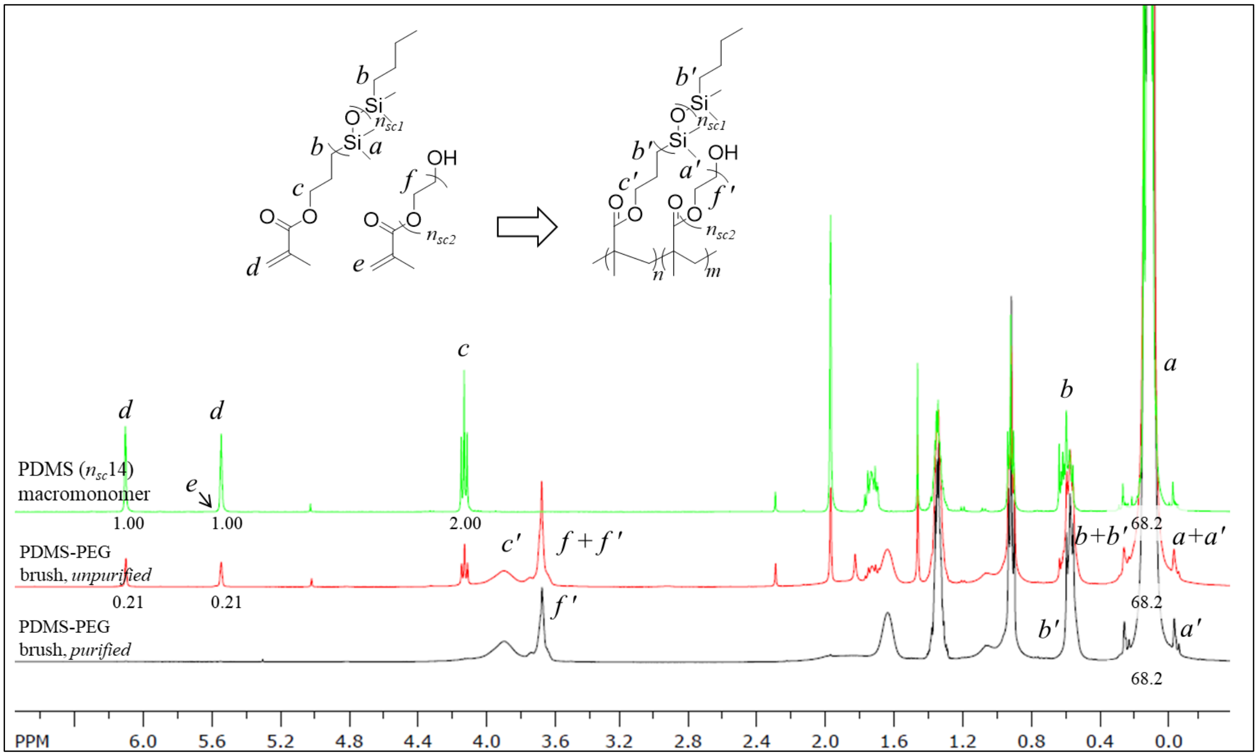

Figure 4. ^{1}H-NMR of random polydimethylsiloxane-poly(ethylene glycol) brushes (PDMS-*r*-PEG, *n*:*m*, 95:5, n_{sc1}: 14, n_{sc2}: 12) at different stages of synthesis (400 MHz, $CDCl_3$): 6.16, 5.57 ($C\mathbf{H}_2$=C(CH_3)C=O, PDMS macromonomer, s, 1H), 4.12 (CO-OC$\mathbf{H}_2$-, PDMS macromonomer, t, 2H), 3.91 (CO-OC$\mathbf{H}_2$-, PDMS brush, t, 2H), 3.78 (CO-OC$\mathbf{H}_2$-, PEG brush, t, 2H), 3.67 (-O$C_2\mathbf{H}_4$O-, PEG brush, m, 32H), 0.55 (-CH_2-(Si(CH_3)$_2$-O)$_n$-C$\mathbf{H}_2$-CH_2-, PDMS macromonomer and brush mixture, m, 4H), 0.09 (-(Si(C$\mathbf{H}_3$)$_2$-O)$_n$-, PDMS macromonomer and brush mixture, s, 68.2H)

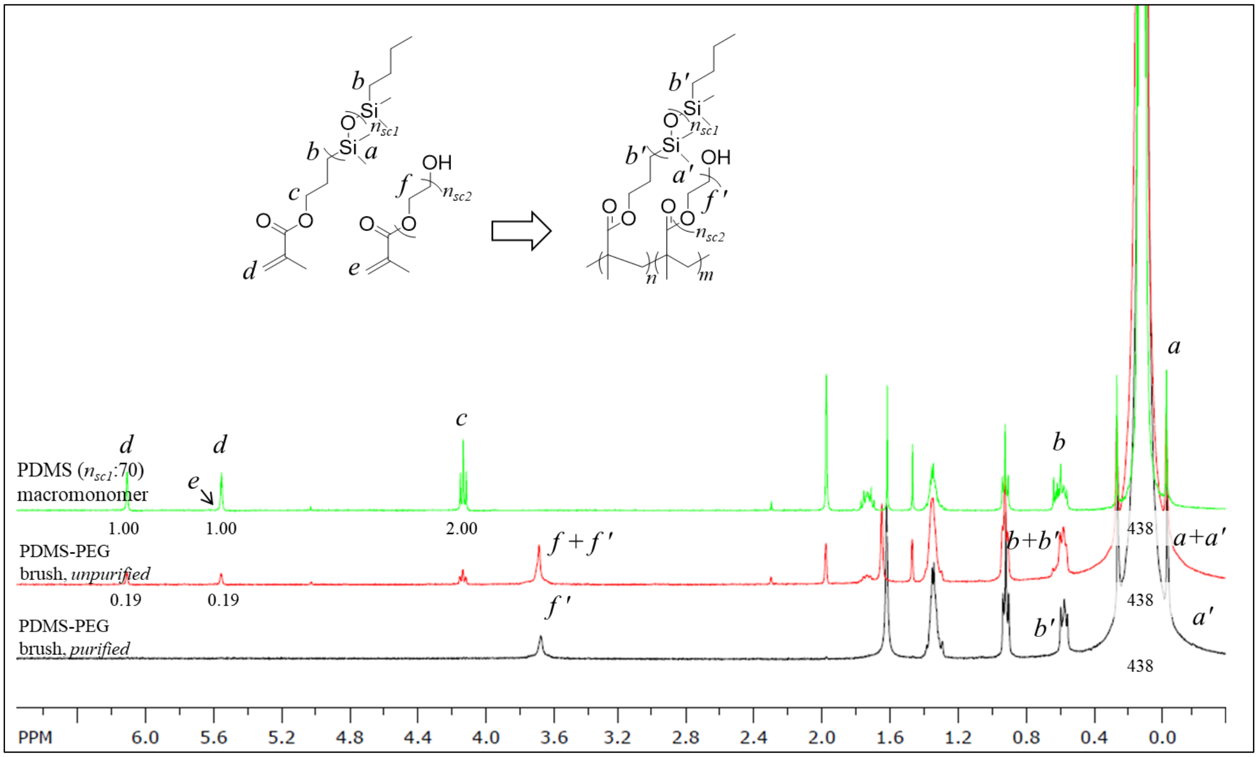

Figure 5. ^{1}H-NMR of random polydimethylsiloxane-poly(ethylene glycol) brushes (PDMS-*r*-PEG, *n*:*m*, 95:5, n_{sc1}: 70, n_{sc2}: 12) at different stages of synthesis (400 MHz, $CDCl_3$): 6.16, 5.57 ($C\mathbf{H}_2$=C(CH_3)C=O, PDMS macromonomer, s, 1H), 4.12 (CO-OC$\mathbf{H}_2$-, PDMS macromonomer, t, 2H), 3.91 (CO-OC$\mathbf{H}_2$-, PDMS brush, t, 2H), 3.78 (CO-OC$\mathbf{H}_2$-, PEG brush, t, 2H), 3.67 (-O$C_2\mathbf{H}_4$O-, PEG brush, m, 32H), 0.55 (-CH_2-(Si(CH_3)$_2$-O)$_n$-C$\mathbf{H}_2$-CH_2-, PDMS macromonomer and brush mixture, m, 4H), 0.09 (-(Si(C$\mathbf{H}_3$)$_2$-O)$_n$-, PDMS macromonomer and bottlebrush mixture, s, 438H). Peak *c′* for brushes with n_{sc1}: 70 do not show on NMR in $CDCl_3$ in contrast to n_{sc3}: 14 brushes. $Conv_{PDMS} = ([Area(a + a')/438] - [Area(d)/1])/[Area(a)/438] = 81\%$. $n_{bb} = Conv_{PDMS} * \frac{[M]}{[I]} = 81\% * 375 = 304$.

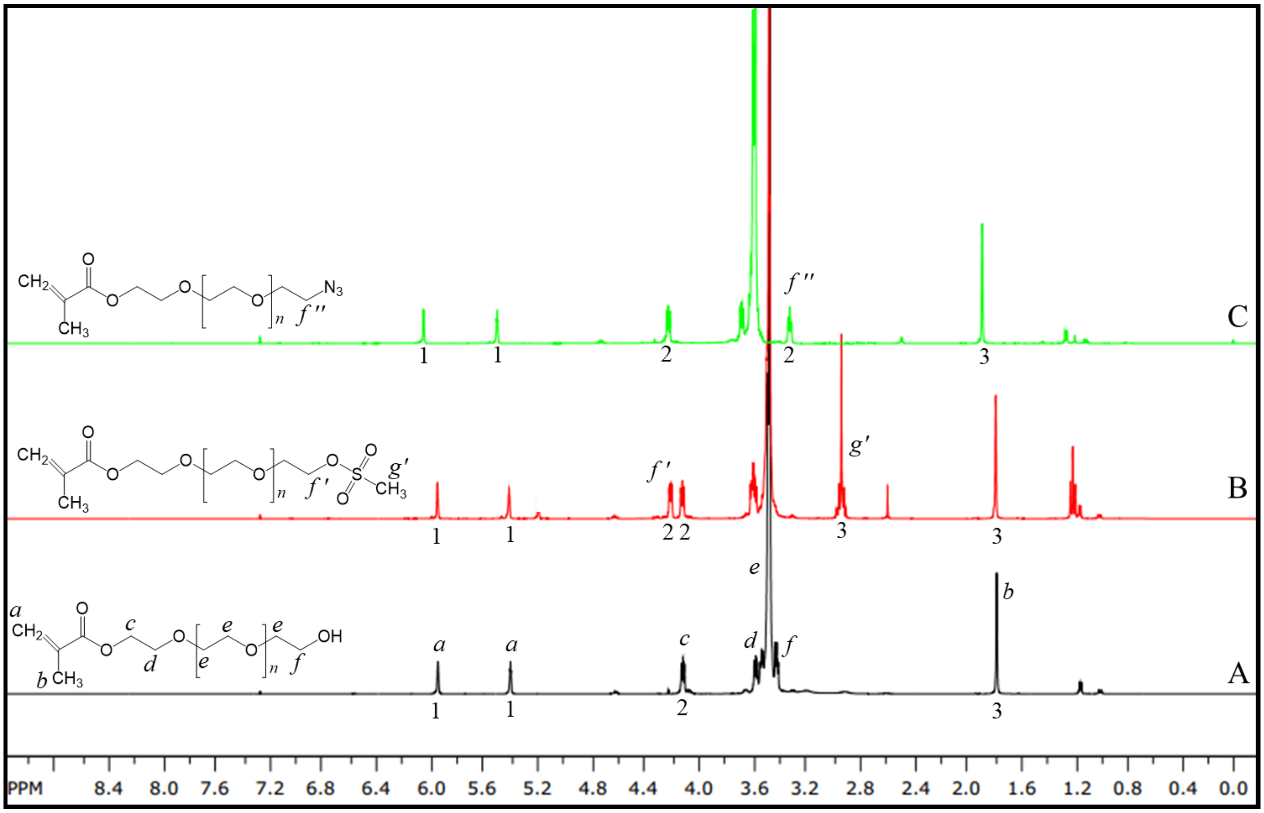

Figure 6. ^{1}H-NMR of poly(ethylene glycol) macromonomer functionalization at different stages. **A**, poly(ethylene glycol) (PEG) macromonomer (400 MHz, $CDCl_3$): 5.98, 5.41 ($C\mathbf{H_2}=C(CH_3)C=O$, s, 1H), 4.15 ($CO\text{-}OC\mathbf{H_2}\text{-}$, t, 2H), 3.59 ($CO\text{-}OCH_2\text{-}C\mathbf{H_2}O\text{-}$, t, 2H), 3.48 ($\text{-}OC_2\mathbf{H_4}O\text{-}$, m, 32H), 3.42 ($\text{-}C\mathbf{H_2}OH$, t, 2H), 1.8 ($CH_2=C(C\mathbf{H_3})C=O$, s, 3H). **B**, PEG macromonomer after mesylation reaction (400 MHz, $CDCl_3$): 5.98, 5.41 ($C\mathbf{H_2}=C(CH_3)C=O$, s, 1H), 4.22 ($\text{-}C\mathbf{H_2}OSO_2CH_3$, t, 2H), 4.15 ($CO\text{-}OC\mathbf{H_2}\text{-}$, t, 2H), 3.59 ($CO\text{-}OCH_2\text{-}C\mathbf{H_2}O\text{-}$, t, 2H), 3.48 ($\text{-}OC_2\mathbf{H_4}O\text{-}$, m, 32H), 2.96 ($\text{-}CH_2OSO_2C\mathbf{H_3}$, s, 3H), 1.8 ($CH_2=C(C\mathbf{H_3})C=O$, s, 3H). **C**, azide-terminated PEG macromonomer (400 MHz, $CDCl_3$): 6.05, 5.52 ($C\mathbf{H_2}=C(CH_3)C=O$, s, 1H), 4.21 ($CO\text{-}OC\mathbf{H_2}\text{-}$, t, 2H), 3.68 ($CO\text{-}OCH_2\text{-}C\mathbf{H_2}O\text{-}$, t, 2H), 3.60 ($\text{-}OC_2\mathbf{H_4}O\text{-}$, m, 32H), 3.37 ($\text{-}C\mathbf{H_2}N_3$, t, 2H), 1.90 ($CH_2=C(C\mathbf{H_3})C=O$, s, 3H).

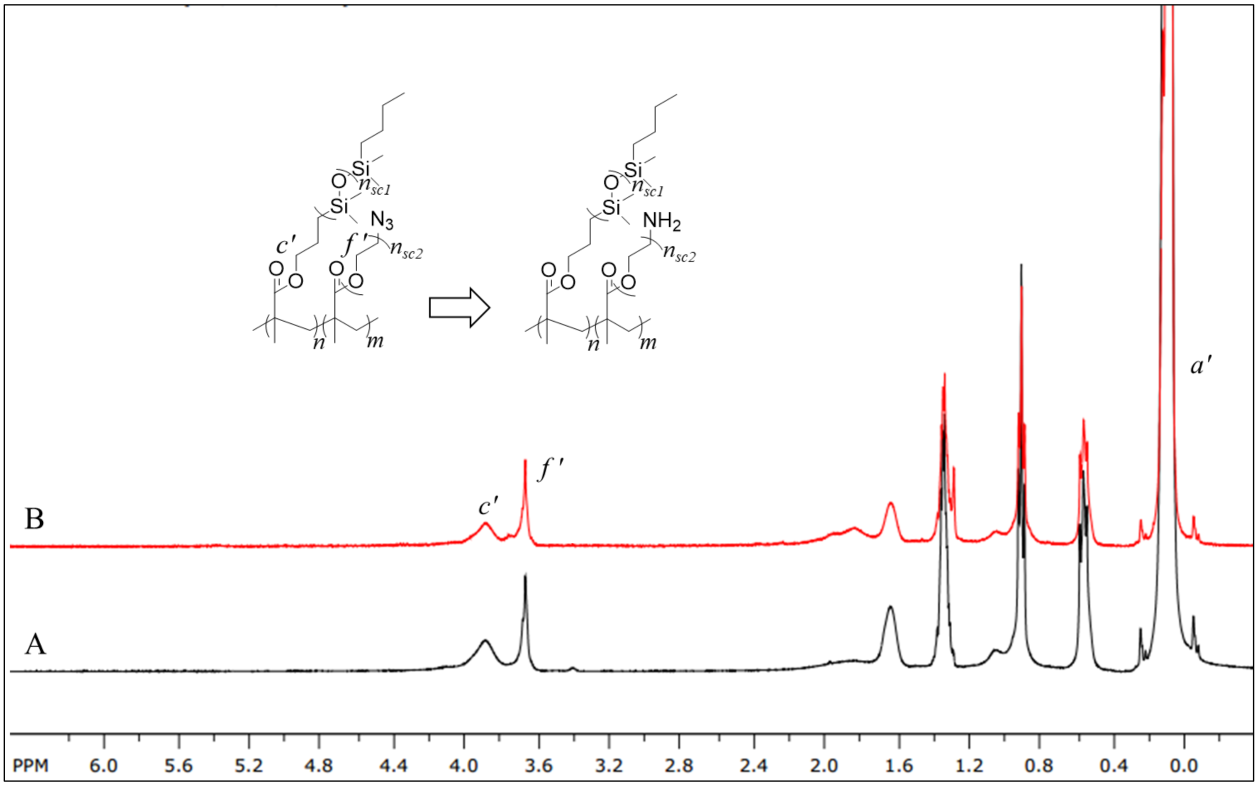

Figure 7. ^{1}H-NMR (400 MHz, $CDCl_3$) of **A**, random polydimethylsiloxane/azide-terminated poly(ethylene glycol) (PDMS-*r*-PEG.N_3), and **B**, random polydimethylsiloxane/amine-terminated poly(ethylene glycol) (PDMS-*r*-PEG.NH_2) bottlebrush copolymer.

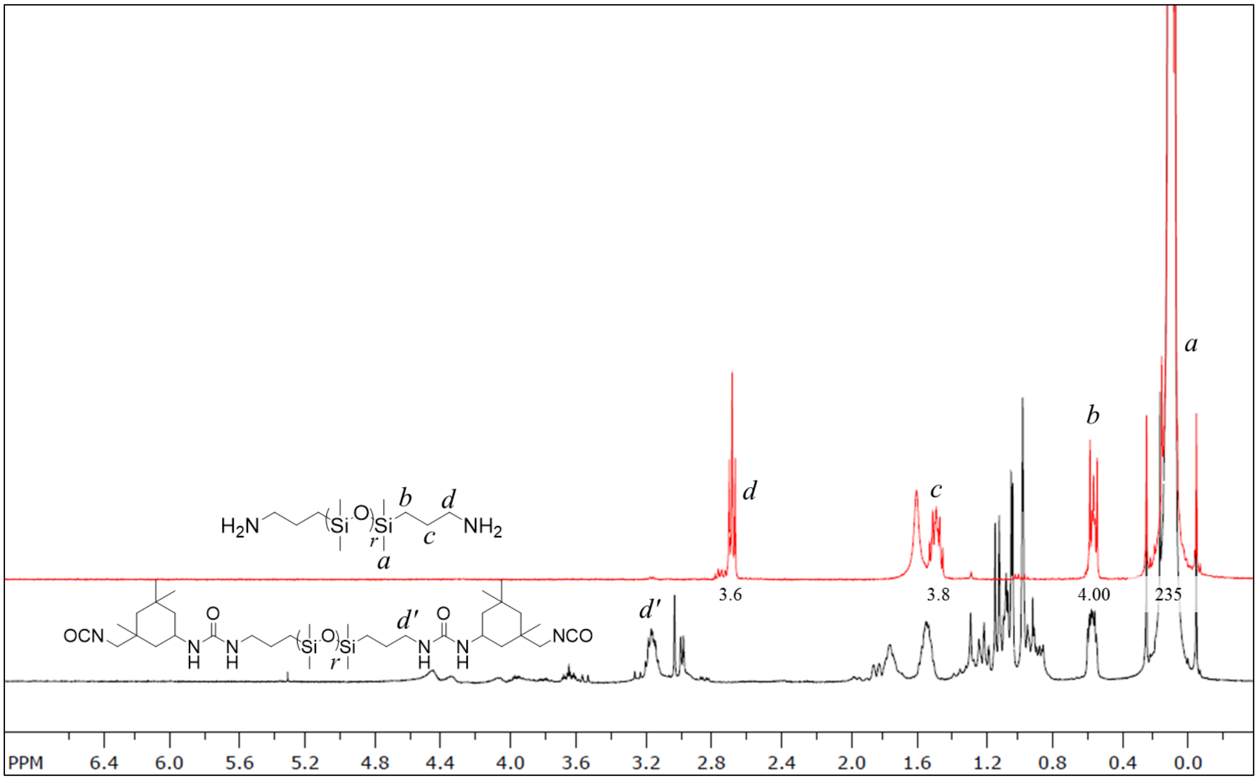

Figure 8. ^{1}H-NMR of polydimethylsiloxane diisocyanate crosslinker (NCO.PDMS.NCO) at different stages of synthesis (400 MHz, $CDCl_3$): 3.18 (-C$\mathbf{H}_2$-NH_2-, crosslinker, t, 2H) 2.69 (-C$\mathbf{H}_2$-NH_2, t, 2H), 0.09 (-(Si(C$\mathbf{H}_3$)$_2$-O)$_n$-, s, 235H).

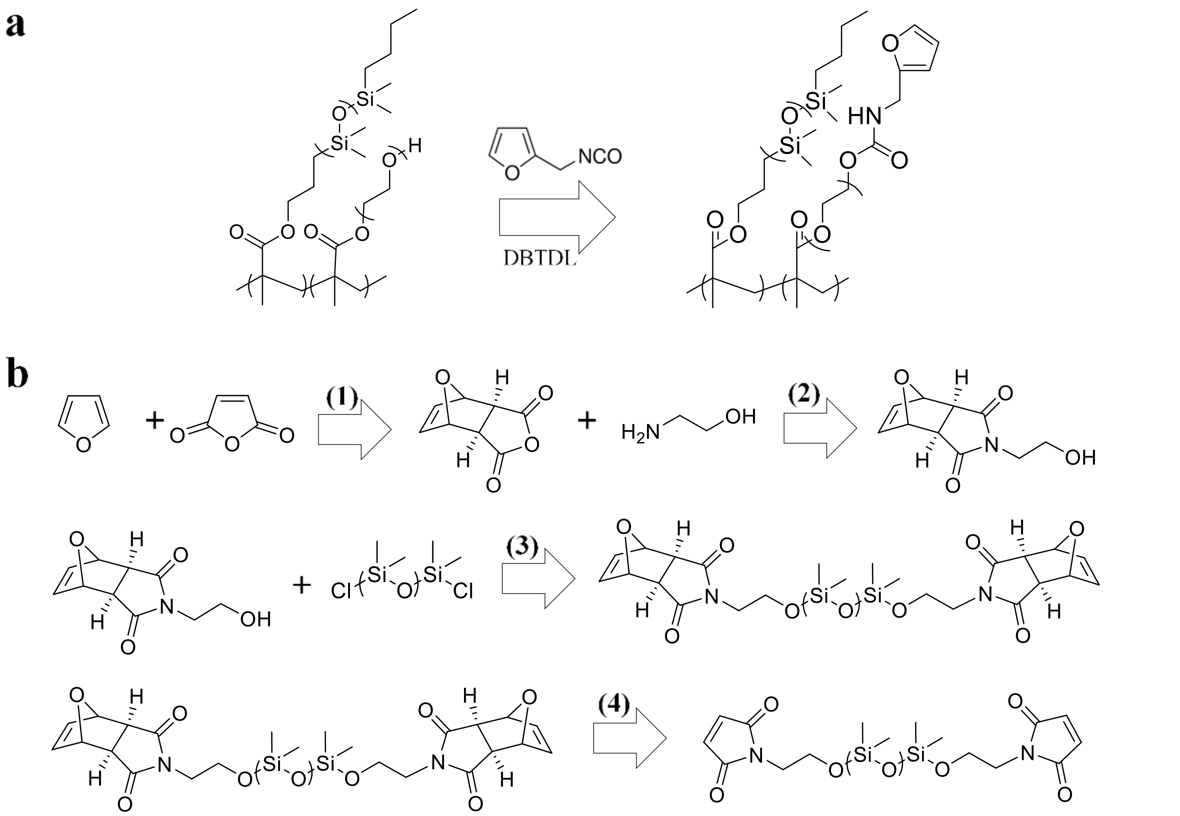

Figure 9. Synthesis of injectable dynamic tissue-mimetic elastomers: **a**, Synthesis of random polydimethylsiloxane-poly(ethylene glycol) (PDMS-*r*-PEG) bottlebrush macromolecules comprising furan moieties. **b**, Synthesis of a linear bifunctional polydimethylsiloxane (PDMS) crosslinker with maleimide moieties: **(1)** synthesis of *exo*-3,6-epoxy-1,2,3,6-tetrahydrophthalic anhydride (furan-protected maleic anhydride), **(2)** synthesis of 2-(2-hydroxyethyl)-3a,4,7,7a-tetrahydro-1H-4,7-epoxyisoindole-1,3(2H)-dione (furan-protected N-(2-hydroxyethyl) maleimide), **(3)** functionalization of chlorine terminated PDMS with furan-protected N-(2-hydroxyethyl) maleimide, **(4)** Maleimide terminated PDMS as linear bifunctional crosslinker for injectable dynamic tissue-mimetic elastomers (for details of illustrated reactions, please see Methods Section).

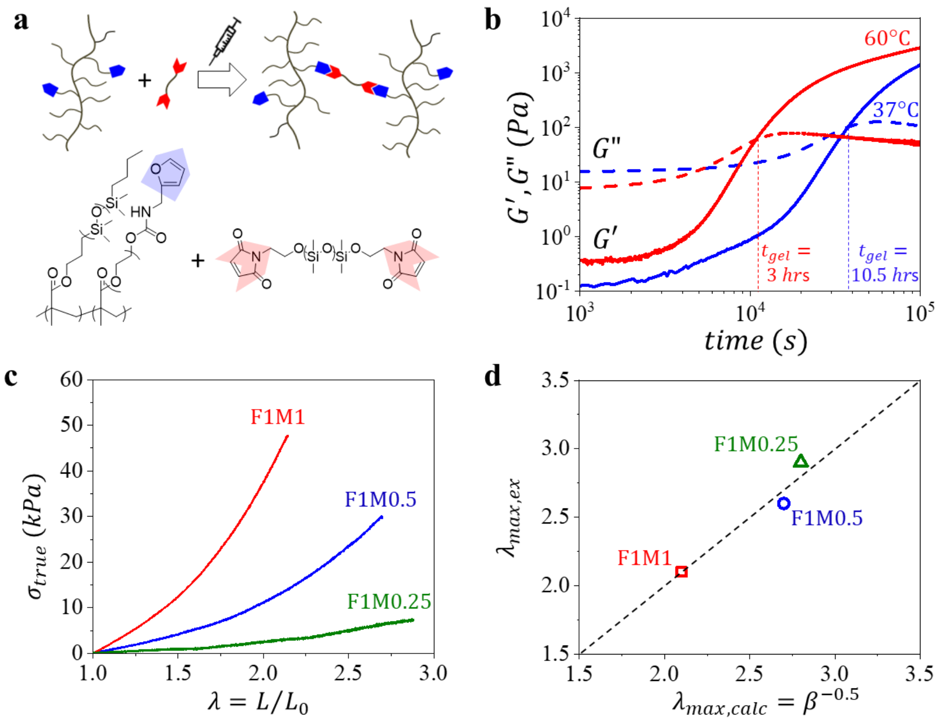

Figure 10. **a**, Injectable reversible tissue-mimetic elastomers composed of random polydimethylsiloxane-poly(ethylene glycol) (PDMS-*r*-PEG) comprising furan (F) moieties with a controlled fraction of a linear bifunctional crosslinker with maleimide (M) moieties (*e.g.*, F1M1 corresponds to 1:1 molar ratio). **b**, Evolution of storage (G′) and loss (G″) moduli as a function of time for injectable dynamic elastomer F1M1 at temperatures of 37 and 60ºC. At 37 ºC, the curing time was about 11h, which enables injection of bulky body implants during time-consuming surgery. **c**, True stress-elongation ($\sigma_{true} - \lambda$) curve profiles of the injectable dynamic tissue-mimetic elastomers. **d**, Experimental elongation-at-break ($\lambda_{max,ex}$) demonstrates good agreement with the maximum strand elongation calculated as $\lambda_{max,calc} = R_{max}/\sqrt{\langle R_{in}^2 \rangle} \equiv \beta^{-0.5}$.

Table 1. Structural and mechanical parameters of injectable dynamic elastomers based on Diels-Alder chemistry (Figure S10c).

F:M [1]	n_{sc} [2]	n_{bb} [3]	n_x [4]	E (kPa) [5]	β [6]	E_0 (kPa) [7]	λ_{max}^{exp} [8]	λ_{max}^{calc} [9]	ϕ_{gel} [10]
F1M1	14	889	50	15.3	0.23	22.3	2.1	2.1	> 98%
F1M0.5	14	889	100	6.3	0.14	7.8	2.7	2.6	> 96%
F1M0.25	14	889	200	1.5	0.12	1.8	2.9	2.8	> 91%

[1] The ratio of furan (F) moieties on PDMS-*r*-PEG bottlebrushes to maleimide (M) moieties on linear bifunctional crosslinker (*e.g.*, F1M1 corresponds to 1:1 molar ratio). Degrees of polymerization (DP) of [2] side-chains and [3] backbone of bottlebrush macromolecules prior to crosslinking determined by ^{1}H-NMR. [4] Nominal DP of the backbone strand between cross-links. [5] Structural Young's modulus (E), and [6] strain-stiffening parameter obtained by fitting stress-strain curves with Equation 1. [7] Young's modulus from Equation 2. [8] Experimental elongation at break. [9] Theoretical elongation at break as $\lambda_{max,calc} = \beta^{-0.5}$. [10] Gel fraction measured by extraction in dichloromethane. The extracted 2-9% fraction is largely composed of unbound brush macromolecules.

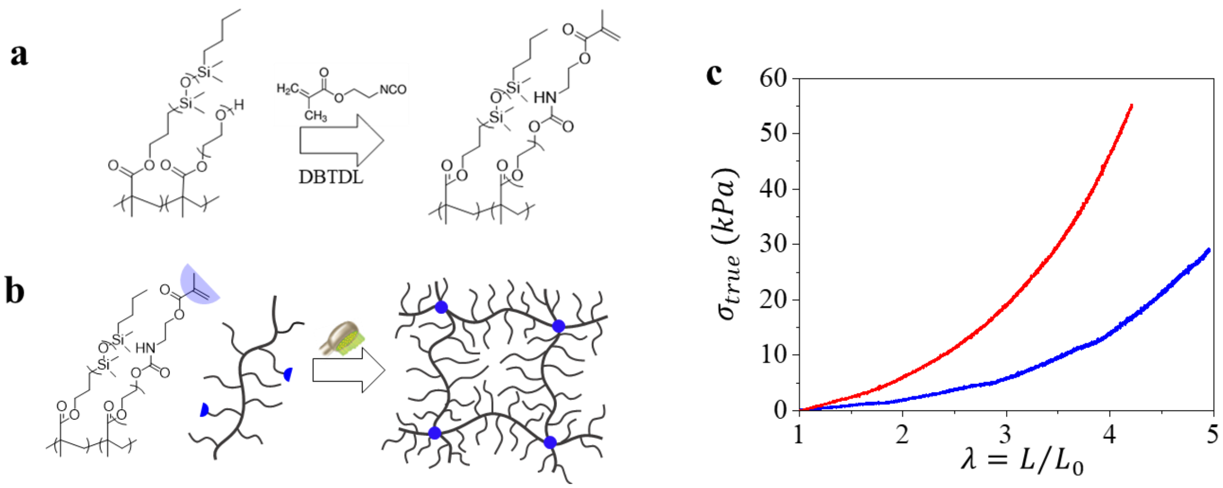

Figure 11. Synthesis of injectable photocurable tissue-mimetic elastomers: **a**, Injectable dynamic tissue-mimetic elastomers composed of random polydimethylsiloxane-poly(ethylene glycol) (PDMS-*r*-PEG) comprising photocurable methacrylate moieties. **b**, True stress-elongation ($\sigma_{true}-\lambda$) curve profiles of the injectable photocurable tissue-mimetic elastomers. The details of conditions of UV procedure are included in the Materials and Methods Section: Functional bottlebrushes were dried with dry N_2 flow until a constant mass was reached. The functionalized brushes were subsequently cured in the presence of diphenyl(2,4,6-trimethylbenzoyl) phosphine oxide/2-hydroxy-2-methylpropiophenone as photo-initiator under N_2 using a UV illumination chamber (365 nm UV lamp, 0.1 mW/cm^{-2}, 10 cm distance).

Table 2. Structural and mechanical parameters of injectable photocurable* elastomers (Figure S11c).

**network** [1)]	n_{sc} [2)]	n_{bb} [3)]	n_x [4)]	E **(kPa)** [5)]	β [6)]	E_0 **(kPa)** [7)]	λ_{max}^{exp} [8)]	λ_{max}^{calc} [9)]	ϕ_{gel} [10)]
Photocure-1.5	14	889	100	4.8	0.06	5.2	4.2	4.1	> 93%
Photocure-3.0	14	889	200	1.7	0.05	1.8	4.9	4.5	> 89%

[1)] Two injectable photocurable tissue-mimetic elastomers are composed of random polydimethylsiloxane-poly(ethylene glycol) (PDMS-*r*-PEG) comprising controlled fraction of PEG macromonomers with chains-end methacrylate moieties at 1.5 and 3 *mol*.%, respectively. Degrees of polymerization (DP) of [2)] side-chains and [3)] backbone of bottlebrush macromolecules prior to crosslinking determined by ^{1}H-NMR. [4)] Nominal DP of the backbone strand between cross-links. [5)]Structural Young's modulus (E), and [6)] strain-stiffening parameter obtained by fitting stress-strain curves with eq 1. [7)] Young's modulus from eq 2. [8)] Experimental elongation at break. [9)] Theoretical elongation at break as $\lambda_{max,calc} = \beta^{-0.5}$. [10)]Gel fraction measured by extraction in dichloromethane. The extracted 2-9% fraction is largely composed of unbound brush macromolecules.

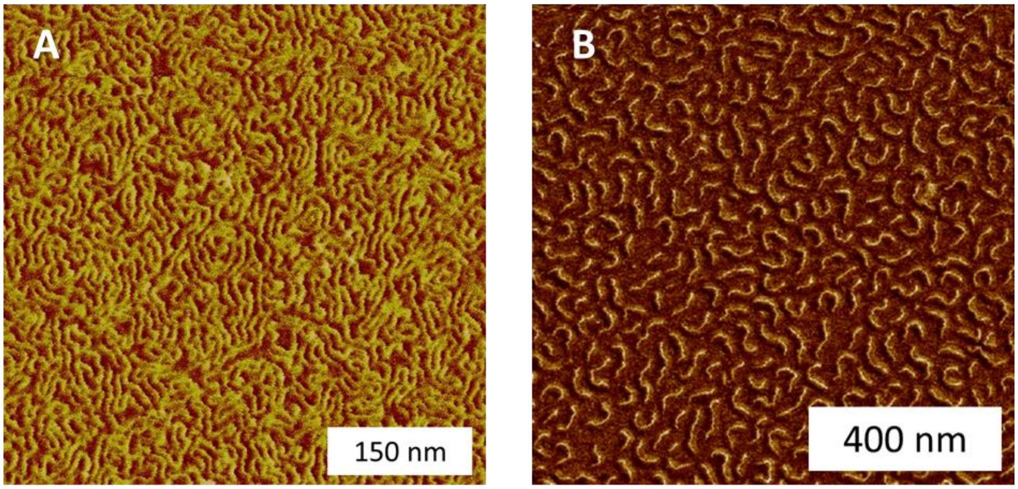

Figure 12. Atomic Force Microscopy of brush polymers. Height micrographs of PDMS-*r*-PEG bottlebrushes deposited on mica by Langmuir-Blodget technique for PDMS: **A**, n_{sc}14, and **B**, n_{sc}70. n_{bb} is determined as L_n/l_0, where L_n is number average measured bottlebrush contour length *via* AFM and l_o= 0.25 nm is the length of bottlebrush backbone monomeric unit. Bottlebrush dispersity, $Đ$ = M_w/M_n is calculated from analysis of > 300 molecules. In coordination with ^{1}H-NMR (Figure. S2-4), AFM adequately and accurately characterizes molecular size dispersity of large macromolecules with branched architecture (Table S1).[1,2]

Table 3. Molecular characterization of PDMS-*r*-PEG bottlebrushes.

Brush Polymer	n_{bb} (NMR)$^{(1)}$	n_{bb} (AFM)$^{(2)}$	$Đ$ (AFM)$^{(3)}$	n_{sc} (AFM)$^{(4)}$
n_{sc}14	889	856±55	1.18	16
n_{sc}70	304	281±35	1.16	63

$^{(1)}$ Number average degree of polymerization of PDMS-*r*-PEG bottlebrush backbone (n_{bb}) determined by ^{1}H-NMR, $^{(2)}$ n_{bb}, $^{(3)}$ length dispersity ($Đ$), and $^{(4)}$side chain degree of polymerization of bottlebrushes determined by AFM (**Figure S12**). n_{bb} was determined by AFM as l_n/l_0, where l_n is number average measured bottlebrush contour length *via* AFM and l_o= 0.25 nm is the length of bottlebrush backbone monomeric unit. Contour length was measured *via* in-house software. Bottlebrush dispersity, $Đ$ = M_w/M_n was calculated based on analysis of ensembles of > 300 molecules to ensure standard deviation of the mean < 10%. n_{sc} was determined by AFM as $l_n/2l_0$ where l_n is number average length between backbones via AFM and l_o= 0.30 nm is the length of PDMS side chain monomeric unit.

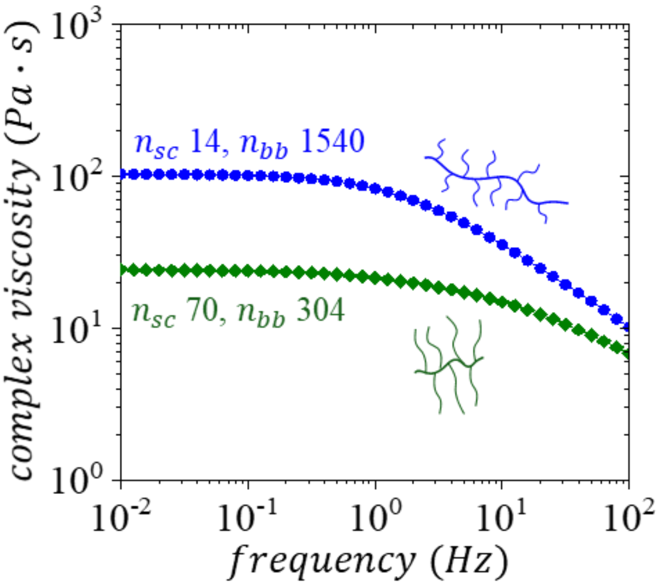

Figure 13. Polydimethylsiloxane (PDMS) bottlebrushes with longer side chains, yet similar molecular weight (M_w = 1,540,000: n_{sc}14, n_{bb}1540 *vs.* M_w = 1,520,000: n_{sc}70, n_{bb}304) possess lower melt viscosity.

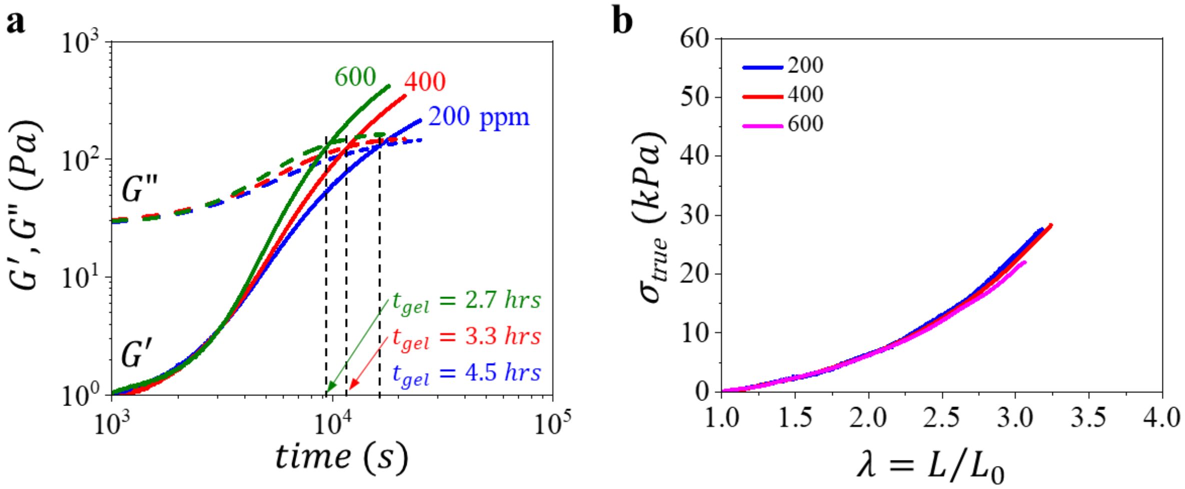

Figure 14. Decoupling gelation time (t_{gel}) and tissue-mimetic mechanics of solvent-free supersoft injectable elastomers: **a**, Evolution of storage (G′) and loss (G″) moduli as a function of time for injectable elastomers comprising NCO:OH ratio 1:4 at different content of catalyst (200, 400, and 600 ppm). **b**, True stress-elongation (σ_{true}– λ) curve profiles of the injectable supersoft solvent-free elastomer comprising NCO:OH ratios 1:4 at different content of catalyst (200, 400, and 600 ppm).

Table 4. Structural and mechanical parameters of NCO:OH (1:4) injectable elastomers* comprising different content of catalyst (200, 400, and 600 ppm (Figure. S16b).

Catalyst [1)]	n_{sc} [2)]	n_{bb} [3)]	n_x [4)]	E (kPa) [5)]	β [6)]	E_0 (kPa) [7)]	λ_{max}^{exp} [8)]	λ_{max}^{calc} [9)]
200	14	889	200	4.32	0.104	5.03	3.18	3.10
400	14	889	200	4.35	0.097	5.00	3.24	3.21
600	14	889	200	4.23	0.095	4.85	3.06	3.24

[1)] Catalyst content (DBTDL, ppm). Degrees of polymerization (DP) of [2)] side-chains and [3)] backbone of bottlebrush macromolecules prior to crosslinking determined by ^{1}H-NMR. [4)] Nominal DP of the backbone strand between cross-links. [5)]Structural Young's modulus (E), and [6)] strain-stiffening parameter obtained by fitting stress-strain curves with Equation 1. [7)] Young's modulus from Equation 2. [8)] Experimental elongation at break. [9)] Theoretical elongation at break as $\lambda_{max,calc} = \beta^{-0.5}$. *The gel fraction of injectable elastomers was > 97%.

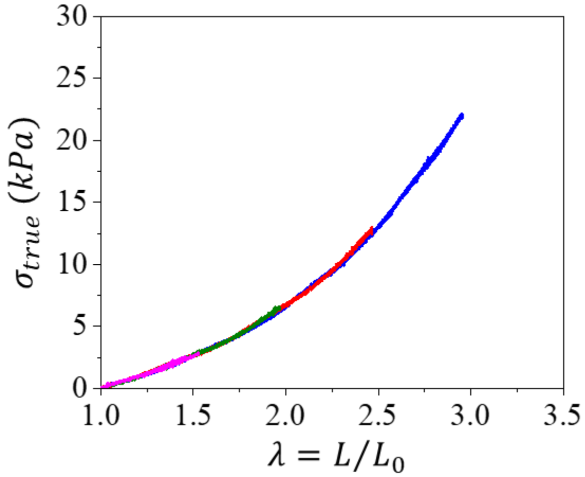

Figure 15. Cyclic loading-unloading curves of injectable elastomer prepared with NCO:OH molar ratio of 1:4 at elongation of λ =1.5 (pink), 2 (green), 2.5 (red), and 3 (blue).

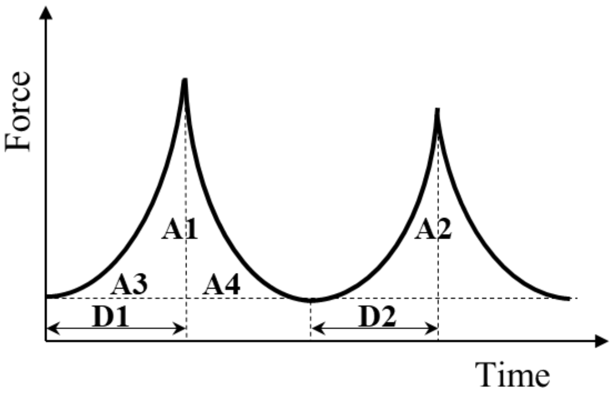

Figure 16. Schematic representation of determining the textural properties including springiness (D2/D1), resilience (A4/A3), and cohesiveness (A2/A1) of injectable non-leaching tissue-mimetic elastomers and commercial implants composed of silicone gel. Texture profile analysis was conducted based on a double compression test.

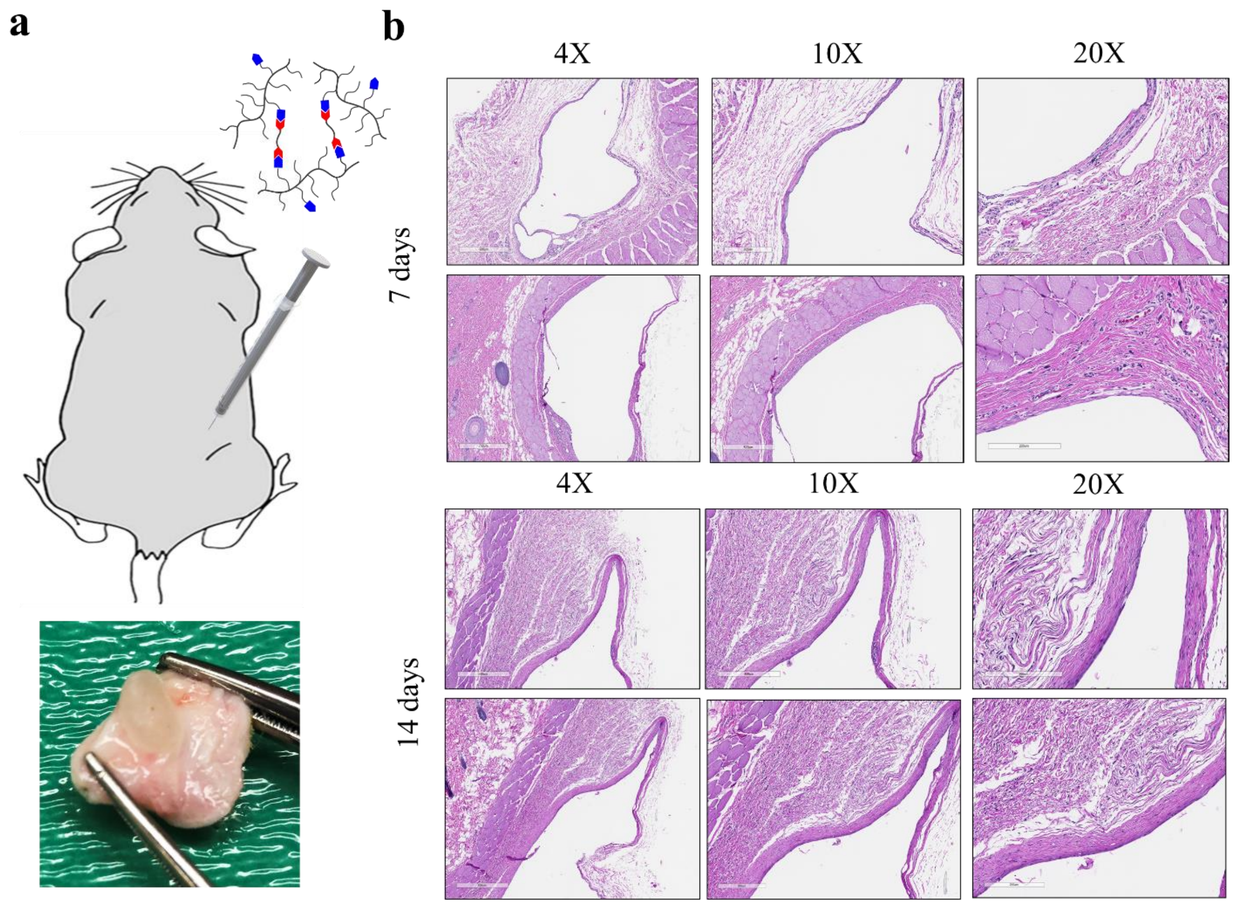

Figure 17. Characterization of direct injection of elastomers *in vivo*. **a**, Schematic and explanted specimen of the injectable elastomer NCO:OH 1:8 after 7 days (top), and 14 days (bottom) after subcutaneous injection. As can be seen, the injectable elastomer does not disperse into the surrounding tissues and is localized in the injection site without any fragmentation. **b**, Histology of subcutaneous injections of elastomer NCO:OH 1:8 at 7, and 14 days explanation stained with hematoxylin and eosin. The histology study showed the slight to moderate inflammatory response without necrosis. The analysis showed a transient inflammatory response with a normal healing process around the injected elastomer.

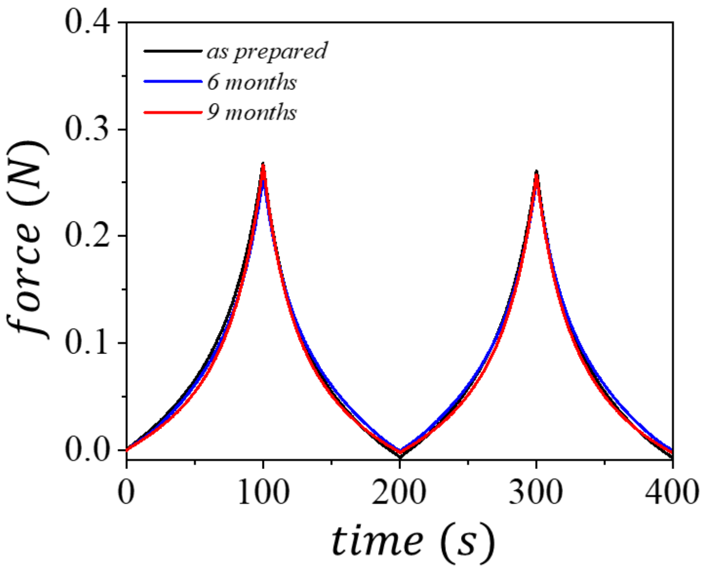

Figure. 18. Comparison of the texture profile analysis of injectable elastomer NCO:OH 1:8 at strain ratio of 50% as prepared, after 6 and 9 months implantation *in vivo*.

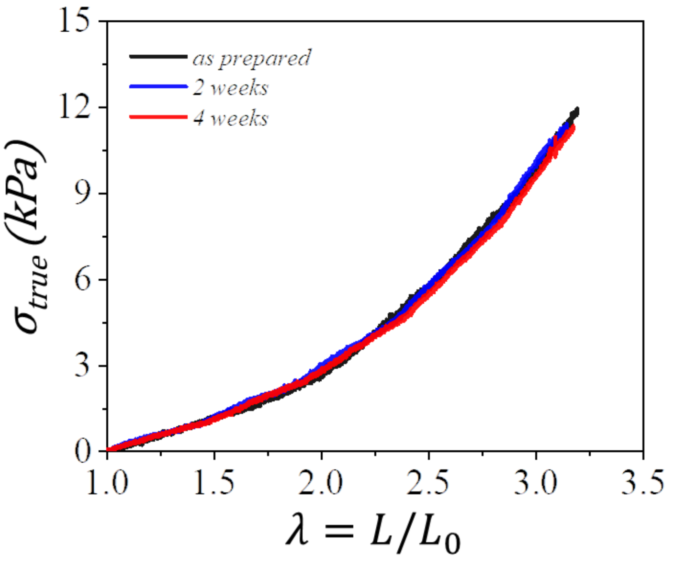

Figure. 19. Comparison of the true stress vs. elongation curves of injectable elastomer NCO:OH 1:8 as prepared, after 2 and 4 weeks incubation in phosphate buffered saline, pH 7.4 at 70ºC.

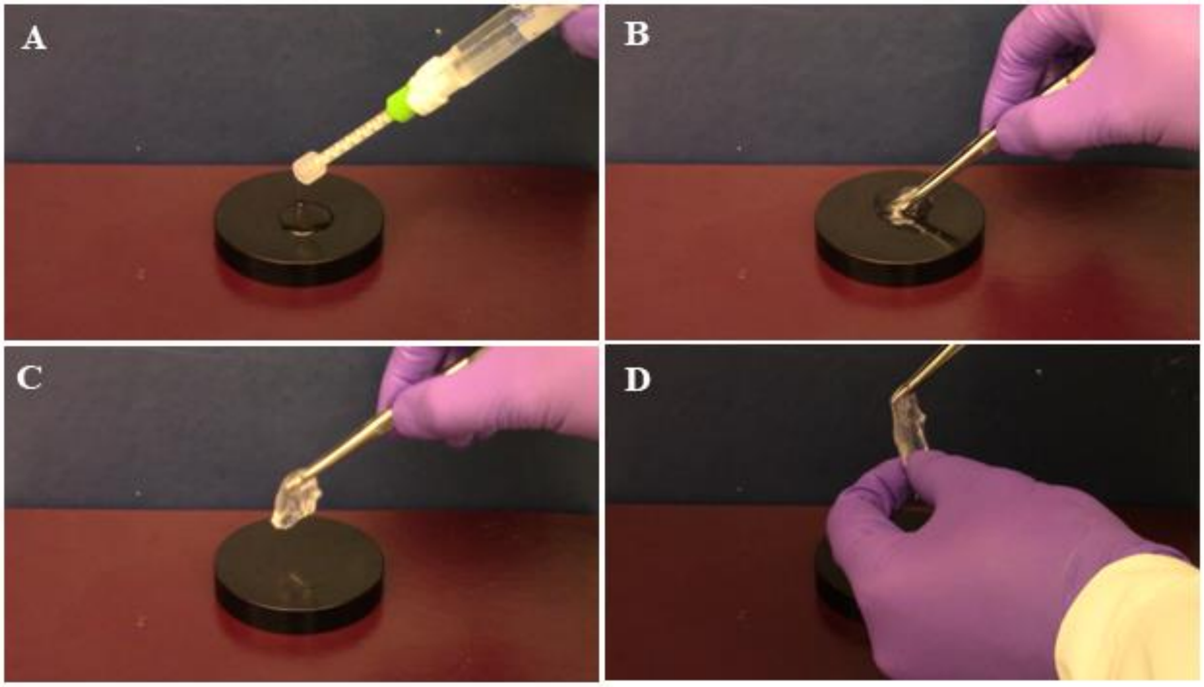

Figure 20. Injectable elastomers: **A**, double-syringe injection, **B**, curing at room temperature, **C**, handling, and **D**, supersoft tissue-mimetic mechanics (Supplementary Video 1).

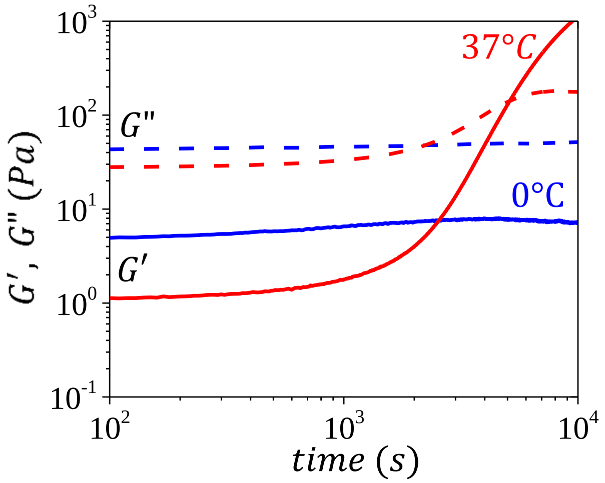

Figure 21. Evolution of elastic (G′) and loss (G″) moduli as a function of time for injectable elastomers composed of brush chains with hydroxyl groups cured with a macromolecular diisocyanate crosslinker NCO:OH (1:1) at temperatures of 0 and 37ºC. The premixed injectable formulation shows gelation at elevated temperature (37ºC), while it remains fluid at low temperature (0ºC). The formulation remained fluid after 2 months storage at -20ºC, and showed gelation with increasing temperature.

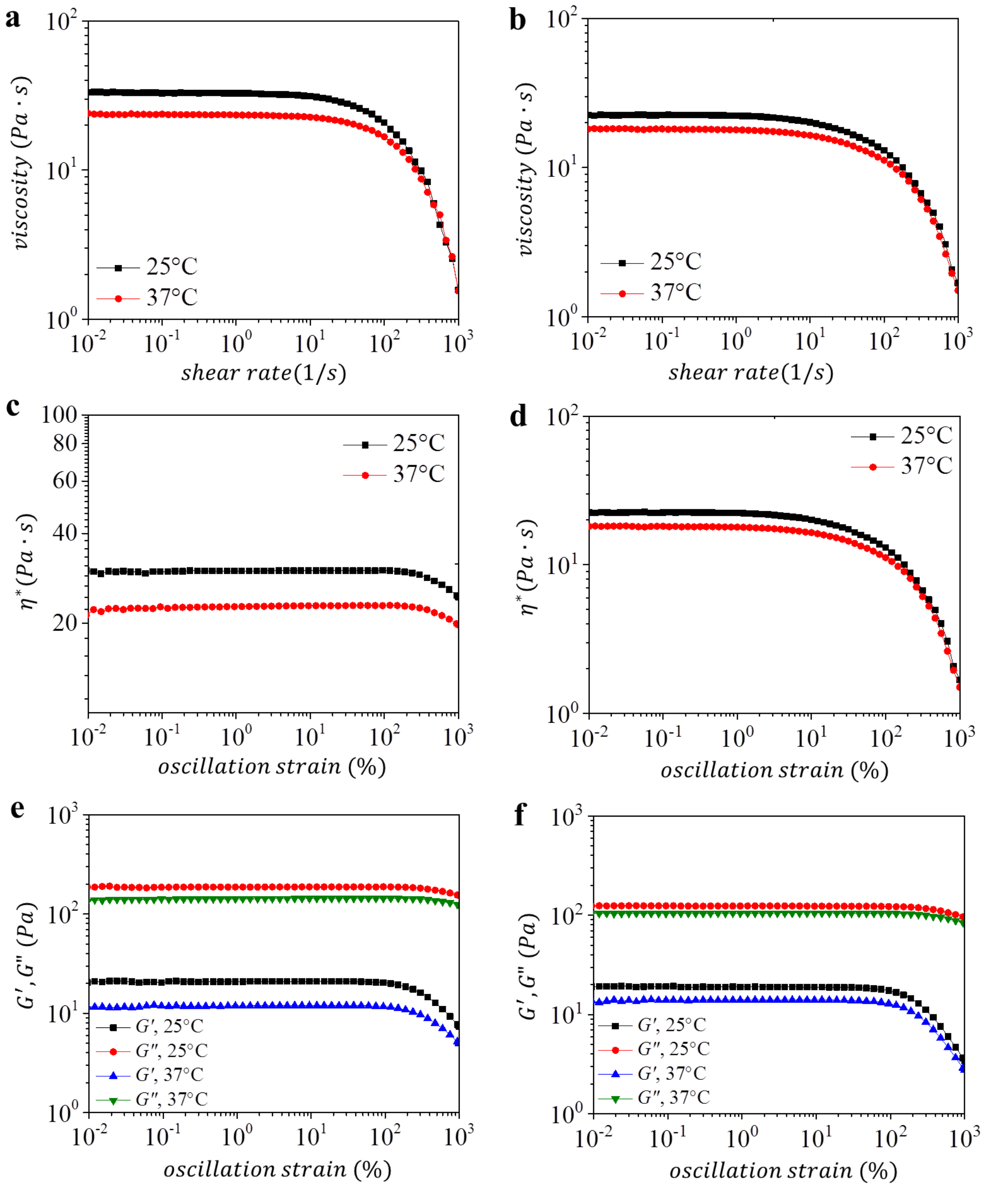

Figure 22. Rheological characterization of random polydimethylsiloxane-poly(ethylene glycol) (PDMS-*r*-PEG) bottlebrush melts as the precursor of injectable elastomers with tissue-mimetic mechanics. Viscosity as a function of shear rate for bottlebrushes with **a**, n_{sc}14, n_{bb}889, and **b**, n_{sc}70, n_{bb}304 at 25 and 37°C. Complex viscosity as a function of oscillation strain at frequency of 1 Hz for bottlebrushes with **c**, n_{sc}14, n_{bb}889, and **d**, n_{sc}70, n_{bb}304 at 25 and 37°C. Storage (G′) and loss (G″) moduli as a function of oscillation strain at frequency of 1 Hz for bottlebrushes with **e**, n_{sc}14, n_{bb}889, and **f**, n_{sc}70, n_{bb}304 at 25 and 37°C.

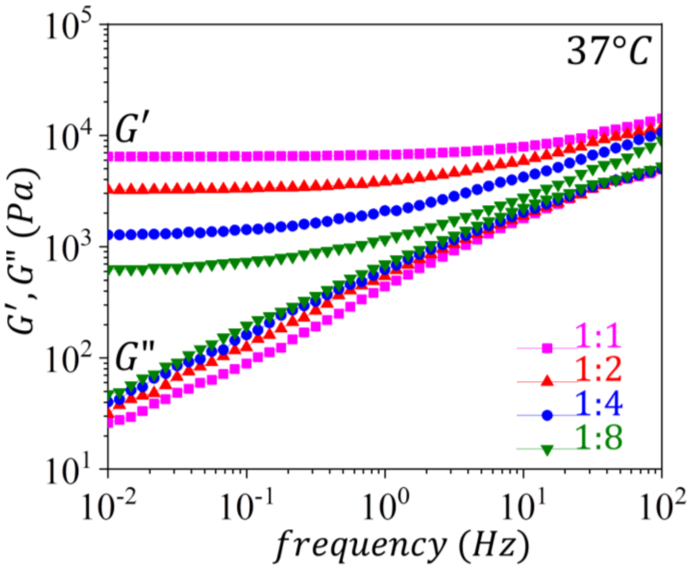

Figure 23. Viscoelasticity of injectable elastomers. Storage (G′) and loss (G″) moduli as a function of frequency for injectable elastomers comprising decreasing NCO:OH ratios (1:1, 2, 4, or 8) at 37°C.

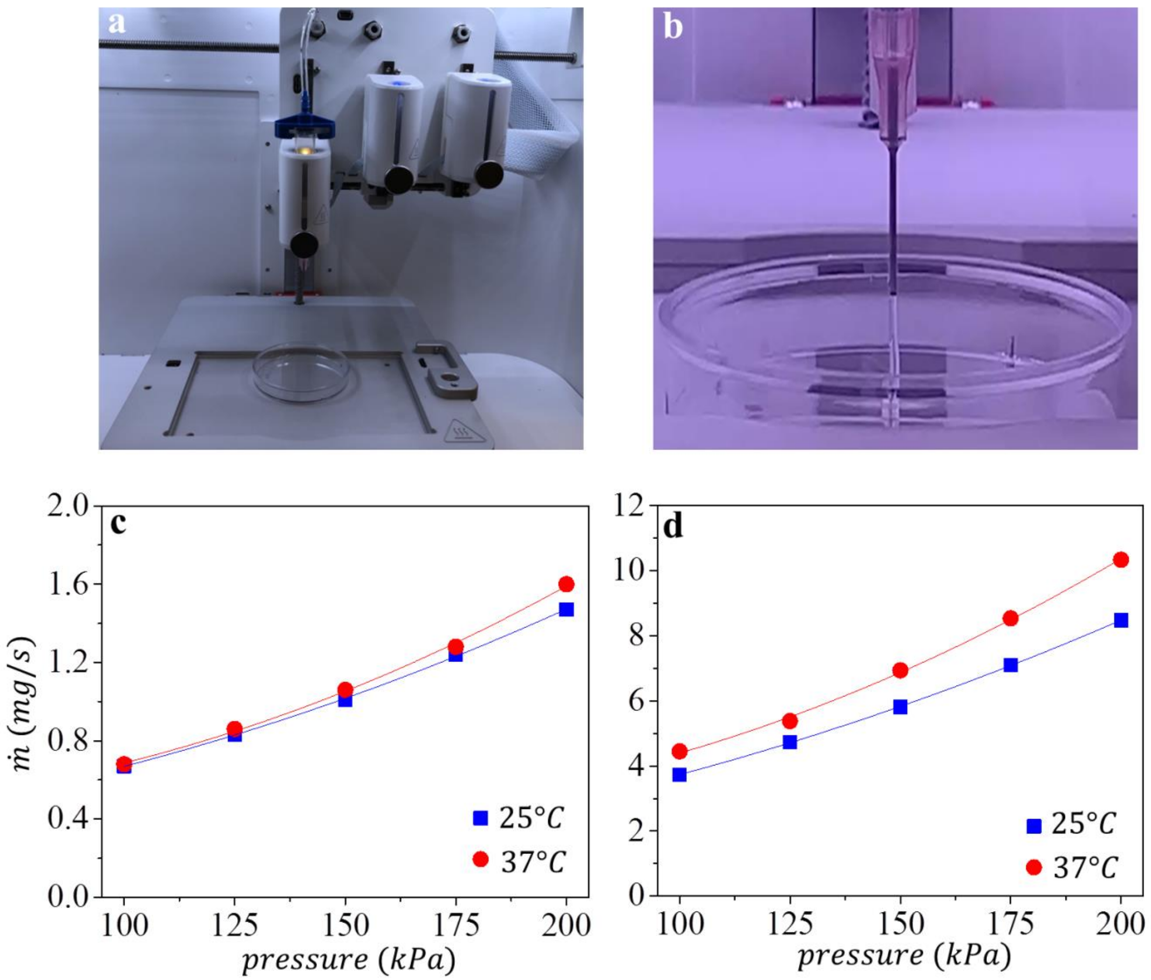

Figure 24. Injectability of random polydimethylsiloxane-poly(ethylene glycol) (PDMS-*r*-PEG) bottlebrush melts as the precursor of injectable elastomers with tissue-mimetic mechanics. **a**, A desktop bioprinter BIO X (CELLINK) with piston-driven syringe heads and pneumatic printheads was used to measure the injectability of bottlebrush melts (Supplementary Video 2). **b**, Injection of PDMS-*r*-PEG bottlebrushes with $n_{sc}14$, $n_{bb}889$ under pressure of 150 kPa at 37°C. Mass flow rate ($\dot{m}$) of PDMS-*r*-PEG bottlebrushes with $n_{sc}14$, $n_{bb}889$ as a function of injection pressure at 25 and 37°C using **c**, 20G (inner diameter: 0.603 mm), and **d**, 16G (inner diameter: 1.194 mm) needles (The lines in the graphs are guide for the eye).

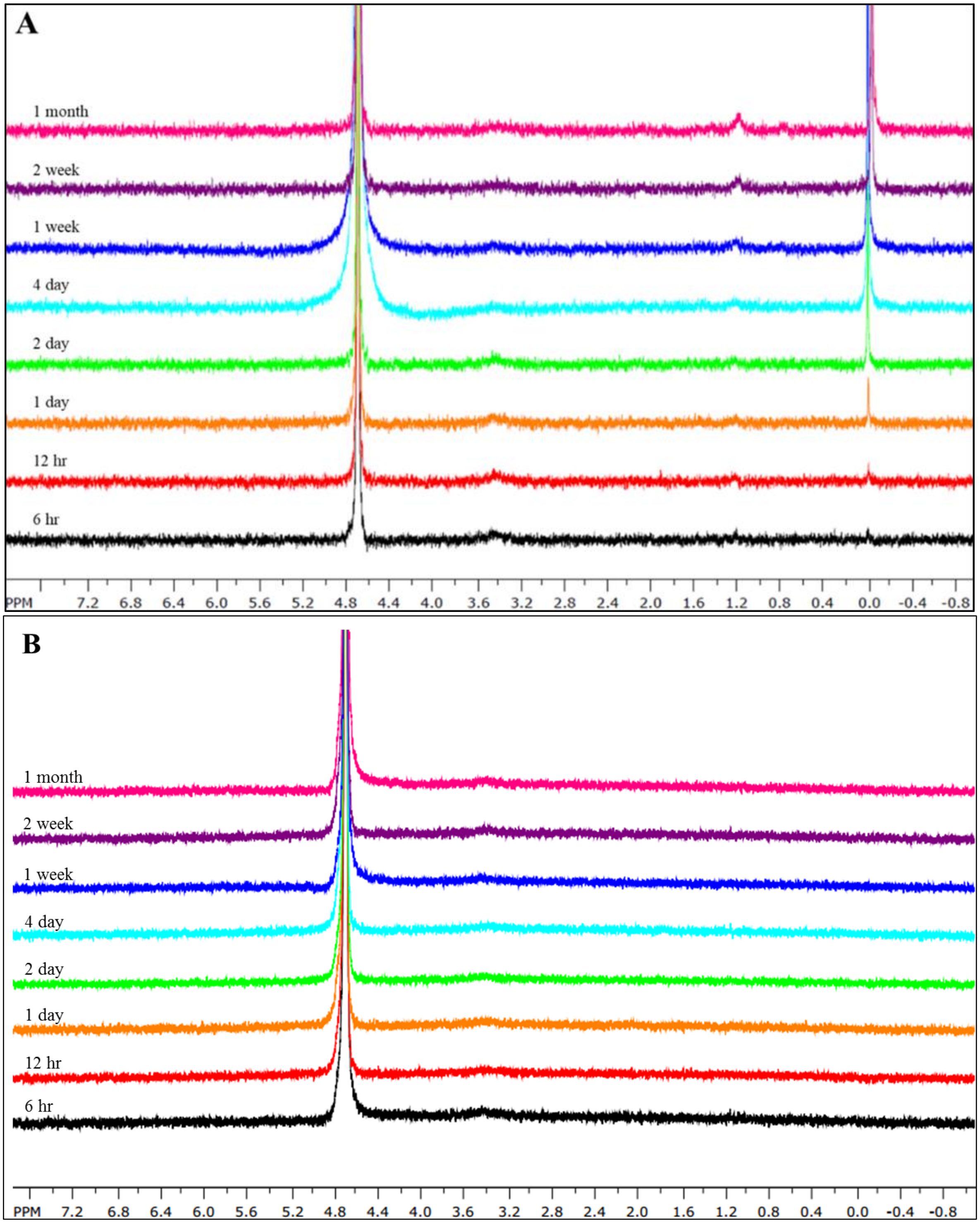

Figure 25. Leachability of injectable elastomer compared to a commercial silicone gel implant into aqueous medium. **A**, Time-resolved ^{1}H-NMR of leachable residue from a commercial silicone gel used in breast implants (*Silicone Gel-1*) in aqueous medium monitored over one month (400 MHz, $CDCl_3$): 4.70 (Residual H_2O), 1.17, 0.01 (leachable materials). **B**, Time-resolved ^{1}H-NMR of leachable residue from a NCO:OH (1:8) injectable elastomer in aqueous medium monitored over a month (400 MHz, D_2O): 4.70 (Residual H_2O); no leachables observed.

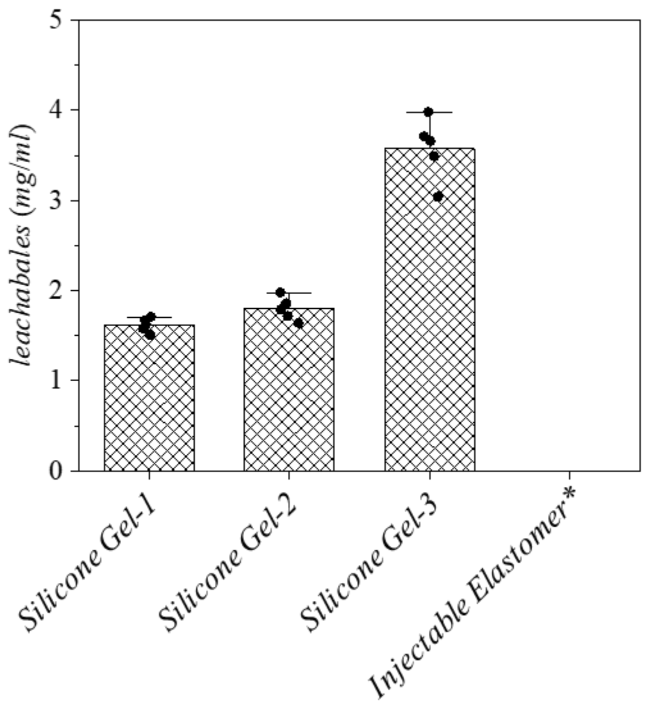

Figure 26. Leachability of three types of commercial silicone gel implants into aqueous medium over a month compared to the injectable elastomer* of NCO:OH (1:8); data shows mass of leachables from 5 gr gel after one month incubation in 10 ml aqueous medium at room temperature. Height of the histogram bins and the error bars correspond to mean values ± SD, respectively. For the leachability test, *n*=5 measurements for independent prepared samples were conducted.

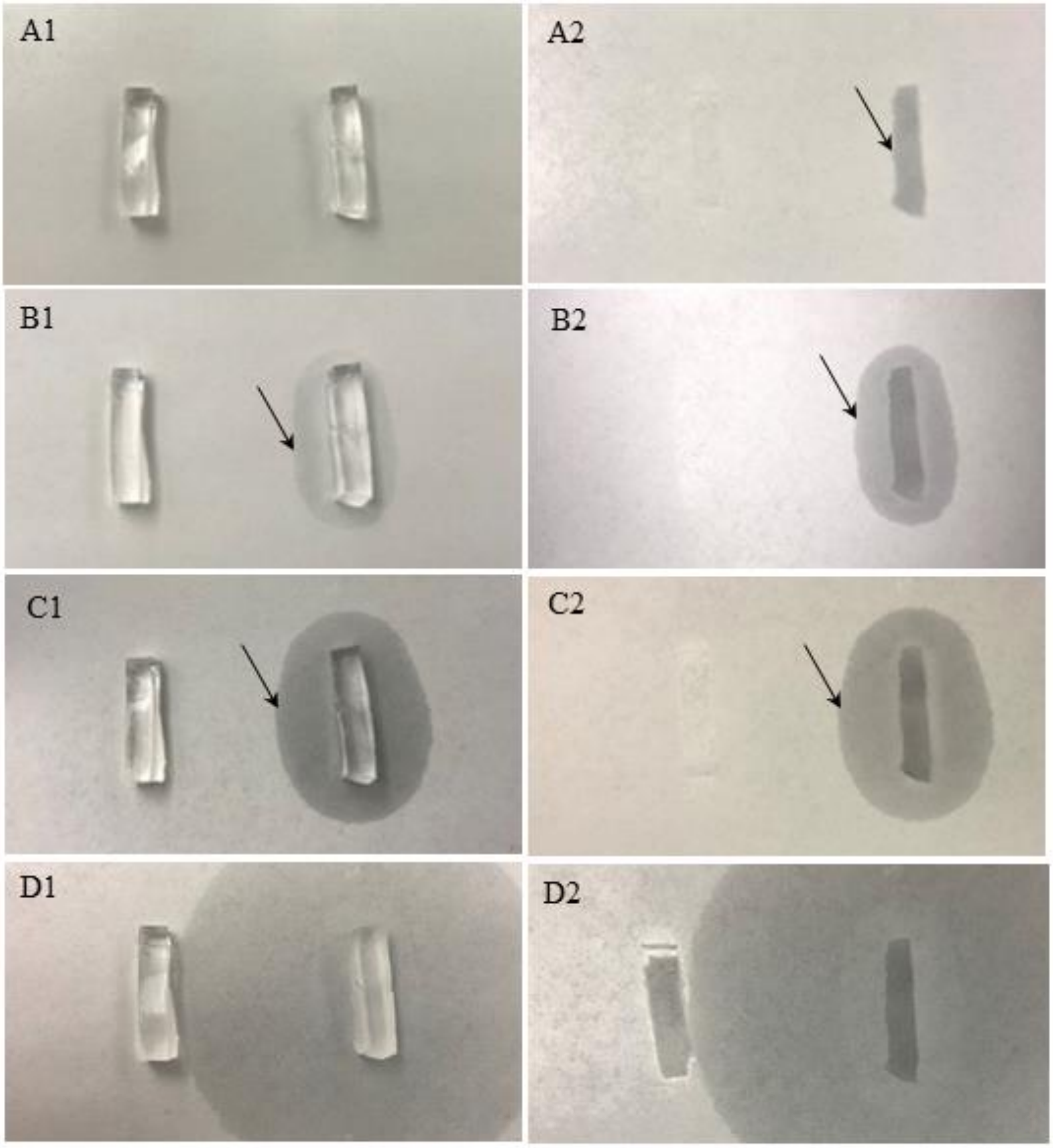

Figure 27. Leachability of a commercial silicone gel used in breast implants (*Silicone Gel-1*) (the right sample in each image) on a paper substrate compared to the injectable elastomer of NCO:OH (1:8) (the left sample in each image). (A1) Front image after 1 hour, (A2) back image after 1 hour, (B1) front image after 1 week, (B2) back image after 1 week, (C1) front image after 1 week, (C2) back image after 1 week, (D1) front image after 1 month, and (D2) back image after 1 month. The leached component from the commercial silicone gel was shown with black arrows.

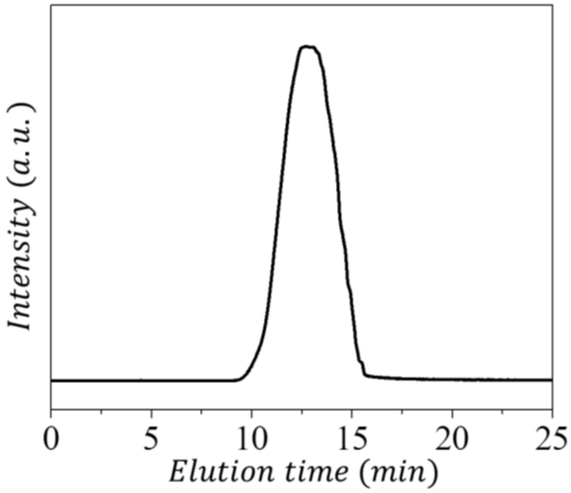

Figure 28. Multi-angle light scattering gel permeation chromatography (MALS-GPC) of random polydimethylsiloxane-poly(ethylene glycol) (PDMS-*r*-PEG) bottlebrushes with n_{sc}14, n_{bb}889: M_n ~ 876 kDa, M_w/M_n=1.006.

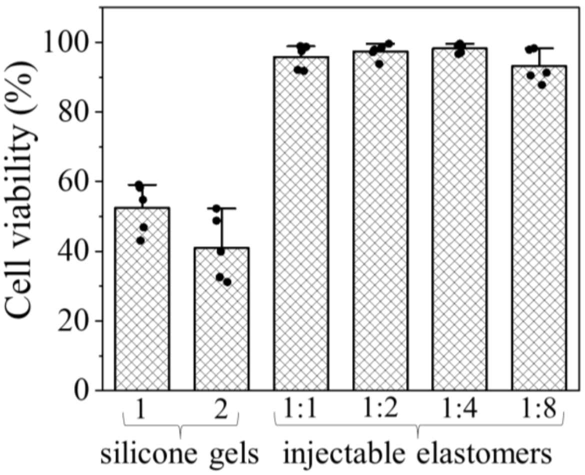

Figure 29. Comparing cytotoxicity of commercial silicone gels and injectable silicone brush elastomers (NCO:OH 1:1→1:8) using human umbilical vein endothelial cells (HUVECs). Height of the histogram bins and the error bars correspond to mean values ± SD, respectively. For the cytotoxicity test, 10^4 cells/cm^2 cells were examined over *n*=5 independent experiments.

References

1 Cong, Y. *et al.* Understanding the synthesis of linear–bottlebrush–linear block copolymers: Toward plastomers with well-defined mechanical properties. *Macromolecules* **53**, 8324-8332 (2020).

2 Sheiko, S. S. *et al.* Measuring molecular weight by atomic force microscopy. *Journal of the American Chemical Society* **125**, 6725-6728 (2003).
