# Supplementary material for: Injectable non-leaching tissue-mimetic bottlebrush elastomers as an advanced platform for reconstructive surgery
Source: Nat Commun. 2021 Jun 25;12:3961. doi: 10.1038/s41467-021-23962-8 (PMC8233362; doi:10.1038/s41467-021-23962-8)
Supplement: Supplementary file 2 — Description of Additional Supplementary Files [file 41467_2021_23962_MOESM2_ESM.docx]

**Description of Additional Supplementary Files**

**Supplementary Video 1.** Demonstration of double-syringe injection, curing at room temperature, handling, and supersoft mechanics of injectable non-leaching tissue-mimetic elastomers.

**Supplementary Video 2.** Demonstrating the injectability of the injectable elastomers. A desktop bioprinter BIO X (CELLINK) with piston-driven syringe heads and pneumatic printheads was used to demonstrate the injectability of the random polydimethylsiloxane-poly(ethylene glycol) (PDMS-rPEG, ݊௦௖14, ݊௕௕889) bottlebrush melt as the precursor of injectable elastomers. Injection was performed under pressure of 150 k
