# Supplementary material for: Injectable non-leaching tissue-mimetic bottlebrush elastomers as an advanced platform for reconstructive surgery
Source: Nat Commun. 2021 Jun 25;12:3961. doi: 10.1038/s41467-021-23962-8 (PMC8233362; doi:10.1038/s41467-021-23962-8)
Supplement: Supplementary file 5 — Reporting Summary [file 41467_2021_23962_MOESM5_ESM.pdf]

nature research

Corresponding author(s): Sergei Sheiko, Mohammad Vatankhah

Last updated by author(s): 2021/04/27

Reporting Summary

Nature Research wishes to improve the reproducibility of the work that we publish. This form provides structure for consistency and transparency in reporting. For further information on Nature Research policies, see our Editorial Policies and the Editorial Policy Checklist.

Statistics

For all statistical analyses, confirm that the following items are present in the figure legend, table legend, main text, or Methods section.

n/a	Confirmed	
☐	☒	The exact sample size (n) for each experimental group/condition, given as a discrete number and unit of measurement
☐	☒	A statement on whether measurements were taken from distinct samples or whether the same sample was measured repeatedly
☒	☐	The statistical test(s) used AND whether they are one- or two-sided<br>*Only common tests should be described solely by name; describe more complex techniques in the Methods section.*
☐	☒	A description of all covariates tested
☐	☒	A description of any assumptions or corrections, such as tests of normality and adjustment for multiple comparisons
☐	☒	A full description of the statistical parameters including central tendency (e.g. means) or other basic estimates (e.g. regression coefficient) AND variation (e.g. standard deviation) or associated estimates of uncertainty (e.g. confidence intervals)
☒	☐	For null hypothesis testing, the test statistic (e.g. F, t, r) with confidence intervals, effect sizes, degrees of freedom and P value noted<br>*Give P values as exact values whenever suitable.*
☒	☐	For Bayesian analysis, information on the choice of priors and Markov chain Monte Carlo settings
☒	☐	For hierarchical and complex designs, identification of the appropriate level for tests and full reporting of outcomes
☒	☐	Estimates of effect sizes (e.g. Cohen's d, Pearson's r), indicating how they were calculated

Our web collection on statistics for biologists contains articles on many of the points above.

Software and code

Policy information about availability of computer code

Data collection	The data were collected using the folloing computer software: TRIOS Version 4.1.1.33073 and Version 4.2.1.36612
Data analysis	The data were analyzed using the Origin 2019 64Nit.Ink software

For manuscripts utilizing custom algorithms or software that are central to the research but not yet described in published literature, software must be made available to editors and reviewers. We strongly encourage code deposition in a community repository (e.g. GitHub). See the Nature Research guidelines for submitting code & software for further information.

Data

Policy information about availability of data

All manuscripts must include a data availability statement. This statement should provide the following information, where applicable:

- Accession codes, unique identifiers, or web links for publicly available datasets
- A list of figures that have associated raw data
- A description of any restrictions on data availability

The authors declare that all data supporting the findings of this study are available within the paper and its supplementary information files. The latter includes full characterization of synthesis and mechanical testing of injectable elastomers reported herein. The respective raw data are available from the corresponding author upon reasonable request.

Field-specific reporting

Please select the one below that is the best fit for your research. If you are not sure, read the appropriate sections before making your selection.

[x] Life sciences [ ] Behavioural & social sciences [ ] Ecological, evolutionary & environmental sciences

For a reference copy of the document with all sections, see nature.com/documents/nr-reporting-summary-flat.pdf

Life sciences study design

All studies must disclose on these points even when the disclosure is negative.

Sample size	The samples size N was determined to ensure a standard deviation of mean below 10% as defined by N=Sum(x_i-x_mean)^2/0.01
Data exclusions	Not data were excluded from data analysis
Replication	All measurements on the same sample were performed in triplicate to ensure reproducibility. All reported data were reproduced.
Randomization	Tests were conducted by one student and then analyzed by a randomly chosen student to ensure bias-free data analysis
Blinding	In critical tests, sample names were not given to students analysing the experimental to maximize the validity of the results

Reporting for specific materials, systems and methods

We require information from authors about some types of materials, experimental systems and methods used in many studies. Here, indicate whether each material, system or method listed is relevant to your study. If you are not sure if a list item applies to your research, read the appropriate section before selecting a response.

Materials & experimental systems

n/a	Involved in the study	
x		Antibodies
	x	Eukaryotic cell lines
x		Palaeontology and archaeology
	x	Animals and other organisms
x		Human research participants
x		Clinical data
x		Dual use research of concern

Methods

n/a	Involved in the study	
x		ChIP-seq
x		Flow cytometry
x		MRI-based neuroimaging

Eukaryotic cell lines

Policy information about cell lines

Cell line source(s)	Fibroblasts (NIH/3T3) and human umbilical vein endothelial cells (HUVECs) were acquired from American Type Culture Collection.
Authentication	Cell lines from ATCC have been thoroughly tested and authenticated: https://www.atcc.org/CellAuthenticationMatters.aspx
Mycoplasma contamination	Both cell lines (NIH/3T3 and HUVECs) tested negative for mycoplasma contamination.
Commonly misidentified lines (See ICLAC register)	N/A

Animals and other organisms

Policy information about studies involving animals; ARRIVE guidelines recommended for reporting animal research

Laboratory animals	Male Wistar white rats with age: 6–7-week-old.
Wild animals	Study did not involve wild animals.
Field-collected samples	The study did not involve samples collected from the field.
Ethics oversight	Animal study was based on a protocol approved by the Institutional Animal Care and Use Committee (IACUC) of The University of North Carolina at Chapel Hill.

Note that full information on the approval of the study protocol must also be provided in the manuscript.
